# Delineating the Association Between Soluble CD26 and Autoantibodies Against G-Protein Coupled Receptors, Immunological and Cardiovascular Parameters Identifies Distinct Patterns in Post-Infectious vs. Non-Infection-Triggered Myalgic Encephalomyelitis/Chronic Fatigue Syndrome

**DOI:** 10.3389/fimmu.2021.644548

**Published:** 2021-04-06

**Authors:** Marvin Szklarski, Helma Freitag, Sebastian Lorenz, Sonya C. Becker, Franziska Sotzny, Sandra Bauer, Jelka Hartwig, Harald Heidecke, Kirsten Wittke, Claudia Kedor, Leif G. Hanitsch, Patricia Grabowski, Nuno Sepúlveda, Carmen Scheibenbogen

**Affiliations:** ^1^ Institute of Medical Immunology, Charité –Universitätsmedizin Berlin, Corporate Member of Freie Universität Berlin, Humboldt-Universität zu Berlin, and Berlin Institute of Health, Berlin, Germany; ^2^ CellTrend, Luckenwalde, Germany

**Keywords:** ME/CFS, chronic fatigue syndrome, CD26, DPP-4, autoantibodies

## Abstract

Soluble cluster of differentiation 26 (sCD26) has a wide range of enzymatic functions affecting immunological, metabolic and vascular regulation. Diminished sCD26 concentrations have been reported in various autoimmune diseases and also in Myalgic Encephalomyelitis/Chronic fatigue syndrome (ME/CFS). Here we re-evaluate sCD26 as a diagnostic marker and perform a comprehensive correlation analysis of sCD26 concentrations with clinical and paraclinical parameters in ME/CFS patients. Though this study did find significantly lower concentrations of sCD26 only in the female cohort and could not confirm diagnostic suitability, results from correlation analyses provide striking pathomechanistic insights. In patients with infection-triggered onset, the associations of low sCD26 with elevated autoantibodies (AAB) against alpha1 adrenergic (AR) and M3 muscarinic acetylcholine receptors (mAChR) point to a pathomechanism of infection-triggered autoimmune-mediated vascular and immunological dysregulation. sCD26 concentrations in infection-triggered ME/CFS were found to be associated with activated T cells, liver enzymes, creatin kinase (CK) and lactate dehydrogenase (LDH) and inversely with Interleukin-1 beta (IL-1b). Most associations are in line with the known effects of sCD26/DPP-4 inhibition. Remarkably, in non-infection-triggered ME/CFS lower sCD26 in patients with higher heart rate after orthostatic challenge and postural orthostatic tachycardia syndrome (POTS) suggest an association with orthostatic regulation. These findings provide evidence that the key enzyme sCD26 is linked to immunological alterations in infection-triggered ME/CFS and delineate a different pathomechanism in the non-infectious ME/CFS subset.

## Introduction

Myalgic encephalomyelitis/chronic fatigue syndrome (ME/CFS) is an acquired and complex chronic disease affecting multiple physical functions. Diagnosis according to Canadian Consensus Criteria includes post exertional malaise (PEM), an exacerbation of symptoms and prolonged period of recovery inadequate to the triggering activity. Further, signs of neurological and autonomous impairment, sleep disturbance, pain, cognitive, gastrointestinal and immunological symptoms are persistent in patients (1). With an estimated prevalence of 0.86 % it goes along with considerable long-term impacts on personal life and health care system (2).

The pathomechanism of ME/CFS is unresolved yet, but there is ample evidence for an autoimmune process involved (3). ME/CFS onset is following an infection in approximately two thirds of patients (4). Various pathogens can trigger ME/CFS with Epstein-Barr virus (EBV) best studied (5). Autoantibodies (AAB) against nuclear and membrane structures as well as neurotransmitter receptors including muscarinic cholinergic receptor M3/M4-antibodies (M3-mAChR/M4-mAChR) and beta-1 and -2-adrenergic receptor (beta1-AR/beta2-AR) have been described in patients with ME/CFS (3, 6–8). These antibodies belong to a network of natural antibodies against adrenergic, cholinergic and other G-protein coupled receptors (GPCR) which were shown to be dysregulated and dysfunctional in various autoimmune diseases (9). Supporting these findings, there is comorbidity of ME/CFS with other autoimmune-associated diseases including Hashimoto’s thyroiditis, Sjogren’s syndrome and inflammatory bowel syndrome (10, 11). Autoimmunity-related risk variants in protein tyrosine phosphatase non-receptor type 22 (*PTPN22*) and cytotoxic T lymphocyte associated protein 4 (*CTLA4*) genes were found to be associated with infection-triggered ME/CFS in a recent study (12). There is no clear evidence that proinflammatory cytokines play a role (13).

Cluster of differentiation 26 (CD26), also known as dipeptidyl peptidase 4 (DPP-4), is a serine protease with a wide range of enzymatic and non-enzymatic functions affecting immune cell activation, vasomotor adaptation and metabolic regulation. CD26 is expressed on the surface of many cells including immune cells. Shedding results in a soluble form consisting of the extracellular part (14). Lymphocytes are considered as the main source of soluble CD26 (sCD26) and release large amounts of pre-stored proteolytically active sCD26 upon activation (15, 16). Cellular CD26 expression on T cells is upregulated upon activation and facilitates T cell co-stimulation, activation and proliferation (17, 18). In line with this, DPP-4 inhibitors were shown to attenuate T cell proliferation (19).

Diminished concentrations of sCD26 were found in various autoimmune diseases including rheumatoid arthritis, Antineutrophil cytoplasmatic antibody (ANCA)-associated vasculitides and inflammatory bowel disease and were shown to correlate negatively with disease activity and inflammatory markers (20–22). In a subset of ME/CFS patients diminished concentrations of sCD26 were reported as well and suggested to be suitable as diagnostic biomarker (23). Two other studies analyzed CD26 surface expression on lymphocytes in ME/CFS. One found higher numbers of CD26+ CD4+ T cells in postviral vs non-viral onset ME/CFS patients (24). In the other study quality of life correlated with CD26 expression levels (25).

In this study we seek to re-evaluate the suitability of sCD26 as a diagnostic marker in ME/CFS patients. Due to the dysregulation of sCD26 in autoimmunity and ME/CFS, further elucidating its association with clinical and laboratory parameters may help to understand its role in ME/CFS. To achieve this, we herein performed a correlation analysis of sCD26 with various clinical and paraclinical parameters to gain further insight into the potential role of sCD26 in ME/CFS.

## Methods

### Human Blood Samples

Patients were diagnosed at the outpatient clinic for immunodeficiencies at the Institute for Medical Immunology at the Charité Universitätsmedizin Berlin. Diagnosis of ME/CFS was based on 2003 Canadian Consensus Criteria and exclusion of other medical or neurological diseases which may cause fatigue. The disease onset with an infection was recorded by patients’ medical history. Healthy controls (HC) were recruited from staff and did not suffer from a disease relevantly impairing their health and physical function. However, neither clinical nor laboratory assessment was performed for controls. The study was approved by the Ethics Committee of Charité Universitätsmedizin Berlin in accordance with the 1964 Declaration of Helsinki and its later amendments. All patients and HC gave informed consent.

### sCD26, Autoantibodies (AAB) and Laboratory Assessment

sCD26 was determined in serum using Human CD26 Platinum ELISA (Thermo Fisher Scientific) according to the manufacturer’s instructions. Alpha1/2 adrenergic receptor (Alpha1-AR/alpha2-AR)-AAB, beta1-AR-/beta2-AR/beta3-AR-AAB, M3-mAChR-/M4-mAChR-AAB as well as Angiotensin-II-receptor type 1 (AT1-R)-AAB and Endothelin receptor A and B (ETA-R/ETB-R)-AAB were determined using ELISA technology by CellTrend GmbH (Luckenwalde, Germany). Routine laboratory values were determined at the Charité diagnostics laboratory (Labor Berlin GmbH, Berlin, Germany). Interleukin 1 (IL-1b) levels were determined in whole blood samples after 4 hours stimulation with lipopolysaccharide (LPS).

### CD26 Surface Expression on Immune Cells

Peripheral blood mononuclear cells (PBMC) from healthy subjects were isolated from heparinized whole blood by density gradient centrifugation and 1x10^6^ PBMCs were stained with viable and dead cells LIVE/DEAD™ Fixable Aqua dead cell stain kit (Life Technologies) for 15 min followed by an extracellular staining. First CCR7 PerCp Cy 5.5 was stained (clone G043H7, Biolegend) for 15 min at 37 °C followed by CD3 AF700 (clone OKT3, Biolegend), CD4 BV605 (clone RPA-T4, BD Biosciences), CD8 PB (clone SK1, Biolegend), CD45 RA FITC (clone HI100, Biolegend), CD56 APC-Cy7 (clone HCD56, Biolegend), CD19 PE-Cy7 (clone HIB19, Biolegend), CD26 APC (clone BA5b, Biolegend) for 15 min at 4°C. Non-specific binding of Fc-receptors was blocked by 2% polyclonal IgG (Flebogamma). CD26 surface expression was measured with CytoFLEX LX (Beckman Coulter) and data was analyzed with FlowJo software 10.0.08. Gating strategies are shown in the **Supplementary Information**.

### Questionnaires for Symptom Assessments

The presence and severity of symptoms in patients with ME/CFS was assessed using a questionnaire based on the 2003 Canadian Consensus Criteria (1, 26). The cardinal symptoms of fatigue, muscle pain, immune symptoms (mean of the 3 symptoms painful lymph nodes, sore throat and flu-like symptoms) and cognitive impairment (mean of the 3 symptoms memory disturbance, concentration ability and mental tiredness) were scored between 1 (no symptoms) and 10 (severest symptoms) by the patients. Symptoms of autonomic dysfunction were assessed by the Composite Autonomic Symptom Score 31 (COMPASS 31) (27). In addition, disability was examined by Bell score focusing on the level of restriction in daily functioning (28) and fatigue using Chalder Fatigue Score (29). Physical activity level of daily life was assessed by Short Form Health Survey (SF-36) (30).

### Statistical Analysis

Statistical data analyses were done using IBM SPSS Statistics 22.0, GraphPad Prism 6.0 and R 4.0. All data were summarized as median with interquartile range (IQR), mean with standard deviation (SD) or number (n) with percentage as appropriate. Comparisons of quantitative parameters between a pair of groups were performed using Mann-Whitney-U rank-sum-test. For comparison of quantitative parameters between age groups Kruskal-Wallis-test with Dunn’s post-hoc analysis was used. Categorical parameters were compared between subgroups applying Chi^2^-test. For evaluation of sCD26 as a diagnostic marker the area under the receiver operating characteristic (ROC) curve was estimated. Correlation analysis was performed using nonparametric Spearman coefficient. Due to multiple testing Benjamini-Yekutieli (BY) correction was applied for each kind of correlated data aiming to control a false discovery rate of 5% while considering possible dependence among parameters. Adjusted p-values <0.05 were considered to provide evidence for a statistically significant result. Significant correlations with sCD26 and their intercorrelations were depicted as network graphs. The network graphs were developed by using Fruchterman-Reingold algorithm from “igraph” package (31). This algorithm was specifically chosen because modeled forces tend to center most frequently used vertices (dots) while less frequent ones are at the outside (Force-directed graph). Bi-directional stepwise multiple regression analysis was performed after log-transformation of data in order to improve criteria of normally distributed residuals. Age and sex were included as possible confounders.

## Results

### Patient Cohort

Concentrations of sCD26 were analyzed in 205 patients with ME/CFS and 98 HC. Median age of patients was 43 (IQR: 33-51) years. 71% of the patients were female and an infection-triggered onset of the disease was reported in 72%. Two patients did not provide information concerning disease onset. Median disease onset was 4 (IQR: 2-9) years before reporting to the clinic. HC were younger than ME/CFS patients by a medium of 14 years (29a (IQR: 26-38)) and 50% were women (**Table 1**). This mismatch was considered in analyses.

**Table 1:** Cohort characteristics and sCD26 concentrations.

	ME/CFS patients			healthy controls (HC)		
	Whole cohort (n: 205)	*Female (n: 145)*	*Male(n: 60)*	Whole cohort (n: 98)	*Female (n: 49)*	*Male (n: 49)*
sCD26 (ng/ml)	648 (IQR: 447-814)	610 (IQR: 382-782)	706 (IQR: 544-882)	670 (IQR: 543-797)	718 (IQR: 594-798)	605 (IQR: 516-796)
age (a)	43 (IQR: 32.5-50.5)	44 (IQR: 33-51)	40.5 (IQR: 30.25-49.75)	29 (IQR: 26-38)	31 (IQR: 27-38)	27 (IQR: 25-37)
infection-triggered onset	n: 146 (71,9%)	n: 105 (72,9%)	n: 41 (69,5%)	—	—	—

Several patients reported comorbidities. 43% of patients suffered from allergies, while 34% reported food intolerances and 66% suffered from symptoms of an irritable bowel syndrome (IBS). Hashimoto’s thyroiditis was present in 11% of the patients, fibromyalgia in 10%. 16% of patients suffered from postural orthostatic tachycardia syndrome (POTS) (for comorbidities see **Supplementary Table S1**).

### sCD26 Concentrations in Patients and HC

**Figure 1:**
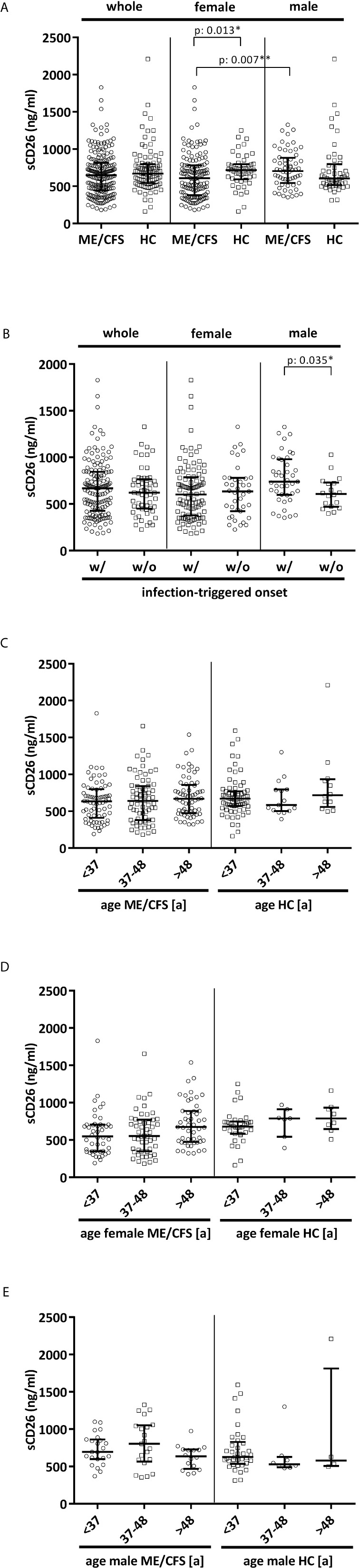
sCD26 in ME/CFS and HC stratified according to sex, disease onset and age. **(A)** ELISA-detected sCD26 serum concentrations in ME/CFS vs. HC and stratified according to sex depicted as boxplots. sCD26 concentrations in sex-groups were compared using Mann-Whitney-U rank-sum test (* = p<0.05; ** = p<0.01). Whole cohort: ME/CFS (n=205): 648 ng/ml (IQR: 447-814) vs. HC (n=98): 670 ng/ml (IQR: 543-797); female cohort: fME/CFSCFS (n=145): 610 ng/ml (IQR: 382-782) vs. fHC (n=49): 718 ng/ml (IQR: 594-798); male cohort: mME/CFS (n=60): 706 ng/ml (IQR: 544-882) vs. mHC (n=49): 605 ng/ml (IQR: 516-796). **(B)** ELISA-detected sCD26 serum concentrations stratified according to disease onset in whole, female and male ME/CFS cohort depicted as boxplots. sCD26 concentrations in onset-groups were compared using Mann-Whitney-U rank-sum test (* = p<0.05; ** = p<0.01). Whole cohort: inf ME/CFS (n=146): 667 ng/ml (IQR: 427-845) vs. non-inf ME/CFS (n=57): 621 ng/ml (IQR: 450-766); female cohort: inf. fME/CFS (n=105): 603 ng/ml (IQR: 377-786) vs. non-inf fME/CFS (n=39): 634 ng/ml (IQR: 422-779); male cohort: inf mME/CFS (n=41): 740 ng/ml (IQR: 597-980) vs. non-inf mME/CFS (n=18): 608 ng/ml (IQR: 471-728). **(C)** ELISA-detected sCD26 serum concentrations age-stratified in ME/CFS and HC. Young age (n=69) was defined as age <34% percentile (<37a), middle age (n=71) as age 34% percentile – 66% percentile (37a-48a) and older age (n=65) as age >66% percentile (>48a) within the cohort of ME/CFS. The same age groups were applied on the HC cohort. sCD26 concentrations were 634 ng/ml (IQR: 411-799) in young age ME/CFS, 640 ng/ml (IQR: 380-843) in middle age ME/CFS and 667 ng/ml (IQR: 474-858) in older age ME/CFS. In young age HC (n=71) sCD26 concentrations were 670 ng/ml (IQR: 572-770), in middle age HC (n= 15) 585 ng/ml (IQR: 503-796) and in older age HC (n=12) 718 ng/ml (IQR: 558-934). Kruskal-Wallis-test to compare groups was not significant (p: 0.357). **(D)** ELISA-detected sCD26 serum concentrations age-stratified in female ME/CFS and HC. Age groups were defined in accordance with **Figure 1C**. Thereby sCD26 concentrations were 549 ng/ml (IQR: 347-705) in young age female ME/CFS (n=46), 553 ng/ml (IQR: 348-771) in middle age female ME/CFS (n=50) and 675 ng/ml (IQR: 474-889) in older age female ME/CFS (n=49). In young female HC (n=34) sCD26 concentrations were 680 ng/ml (IQR: 583-747), in middle age female HC (n=7) 789 ng/ml (IQR: 543-910) and in older age female HC (n=8) 789 ng/ml (IQR: 647-934). Kruskal-Wallis-test was significant at p: 0.009, however, there were no significant differences between subgroups after post-hoc analyses. **(E)** ELISA-detected sCD26 serum concentrations age-stratified in male ME/CFS and HC. Age groups were defined in accordance with **(C)**. Thereby sCD26 concentrations were 695 ng/ml (IQR: 599-861) in young age male ME/CFS (n=23), 803 ng/ml (IQR: 567-1051) in middle age male ME/CFS (n=21) and 635 ng/ml (IQR: 469-727) in older age male ME/CFS (n=16). In young male HC (n=37) sCD26 concentrations were 626 ng/ml (IQR: 534-829), in middle age male HC (n=8) 530 ng/ml (IQR: 488-628) and in older age male HC (n=4) 581 ng/ml (IQR: 507-1813). Kruskal-Wallis-test to compare groups was not significant (p: 0.326).

Overall median sCD26 in ME/CFS patients was 648 ng/ml (IQR: 447-814) and not different from HC with 670 ng/ml (IQR: 543-797). When we stratified our cohort into male and female, sCD26 concentrations were significantly lower in female ME/CFS patients as compared to males (p: 0.007). No difference between sexes was found in HC. Taking this into regard we performed comparisons with HC separately for each sex. Concentrations of sCD26 were significantly lower in female patients than in female HC (p: 0.013), whereas no difference between male patients and HC was found (**Figure 1A**). When stratifying for type of onset sCD26 concentrations were significantly higher in male patients with infection-triggered ME/CFS compared patients without infectious-triggered onset of disease (p: 0.035). Soluble CD26 concentrations did not differ between onset subgroups in female patients (**Figure 1B**). No differences in sCD26 concentrations were seen when patients and HC were divided according to three major age groups (**Figure 1C**). When analyzing age groups of ME/CFS and HC within sexes, overall Kruskal-Wallis test was significant (p: 0.009) in the female group, but none of the differences between age groups remained significant in post-hoc analyses (**Figure 1D**). No significant differences were found in the male group (Kruskal-Wallis p: 0.326; **Figure 1E**).

**Figure 2:**
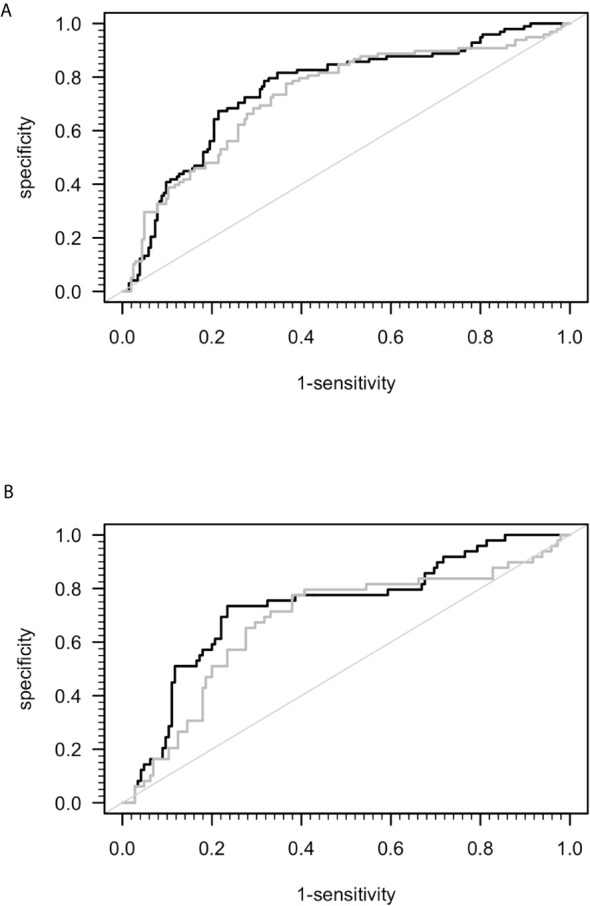
ROC analyses for ME/CFS vs. HC. **(A)** ROC analyses for ME/CFS vs. HC in the whole cohort. Grey ROC curve represents ability of distinction between ME/CFS (n=205) and HC (n=98) only by age and sex (AUC: 0.736, 95% CI: 0.674 – 0.798) and thereby shows the mismatch of patients and controls in this study. Black ROC curve represents ability of distinction after adding ELISA-detected sCD26 serum concentrations to the model (AUC: 0.754, 95% CI: 0.695-0.814). **(B)** ROC analyses for ME/CFS vs. HC in the female cohort. Grey ROC curve represents ability of distinction between ME/CFS (n=145) and HC (n=49) only by age (AUC: 0.687, 95% CI: 0.596-0.779) and thereby shows the mismatch of patients and controls in this study. Black ROC curve represents ability of distinction after adding ELISA-detected sCD26 serum concentrations to the model (AUC: 0.733, 95% CI: 0.649-0.817).

A previous study suggested that sCD26 is a potential diagnostic biomarker for ME/CFS (23). Taking into consideration the mismatch of age and sex between ME/CFS patients and HC in this study, we performed ROC-analyses (**Figure 2**). The area under ROC curve (AUC) of the model of only sex and age for distinguishing ME/CFS from HC (AUC: 0.736, 95% confidence interval (CI): 0.674 – 0.798) did not improve significantly after adding sCD26 (AUC: 0.754, 95% CI: 0.695-0.814) (**Figure 2A**). A similar result was found when aiming to distinguish female patients from female HC by only age (AUC: 0.687, 95% CI: 0.596-0.779) as well as age and sCD26 (AUC: 0.733, 95% CI: 0.649-0.817) (**Figure 2B**). In this study, sCD26 did thereby not demonstrate sufficient diagnostic value.

### Correlation of sCD26 With Clinical Parameters

In order to evaluate effects and interactions of sCD26, we performed correlation analyses with clinical as well as paraclinical parameters. Since deviation in sCD26 concentrations from HC was only seen in female patients, we focused on this group. Patients were stratified according to infection-triggered or non-infection-triggered onset due to the assumed difference in pathomechanism (**Table 2A** shows the significant correlations, all data is shown in **Supplementary Tables S2A–D**).

**Table 2A:** Parameters significantly correlating with sCD26 in female patients (onset stratified).

Parameter	Female ME/CFS cohort (n=145)		Female patients with infection-triggered onset (n=105)		Female patients without infection-triggered onset (n=39)	
	n	Spearman’s r; p	n	Spearman’s r; p	n	Spearman’s r; p
Laboratory assessment:						
IL1b	120	r: -0.171; p: 0.062	83	r: -0.309; p: 0.005*	37	r: 0.095; p: 0576
ACE	120	r: 0.218; p: 0.017*	83	r: 0.177; p: 0.109	37	r: 0.302; p: 0.069
Lymphocytes	131	r: -0.218; p: 0.013*	92	r: -0.249; p: 0.017*	38	r: -0.153; p: 0.358
Monocytes	131	r: -0.181; p: 0.039*	92	r: -0.170; p: 0.105	38	r: -0.178; p: 0.285
CD3+ T cells	131	r: -0.214; p: 0.014*	92	r: -0.207; p: 0.048*	38	r: -0.202; p: 0.223
CD4+ T cells	131	r: -0.231; p: 0.008*	92	r: -0.221; p: 0.034*	38	r: -0.268; p: 0.104
CD4+CD8+ T cells	131	r: 0.230; p: 0.008*	92	r: 0.270; p: 0.009*	38	r: 0.127; p: 0.448
HLA-DR/CD8+ T cells	122	r: 0.312; p: <0.001*	85	r: 0.299; p: 0.005*	37	r: 0.297; p: 0.074
CD11a+/CD8+ T cells	120	r: 0.221; p: 0.015*	83	r: 0.179; p: 0.105	37	r: 0.320; p. 0.054
IgA	143	r: -0.112; p: 0.181	103	r: 0.007; p: 0.947	39	r: -0.366; p: 0.022*
LDH	143	r: 0.121; p: 0.149	103	r: 0.228; p: 0.020*	39	r: -0.149; p: 0.364
CK	138	r: 0.268; p: 0.001*	100	r: 0.329; p: 0.001*	38	r: 0.116; p. 0.487
GPT	143	r: 0.197; p: 0.018*	103	r: 0.271; p: 0.006*	39	r: 0.044; p: 0.792
GOT	143	r: 0.112; p: 0.182	103	r: 0.252; p: 0.010*	39	r: -0.223; p: 0.172
GGT	143	r: 0.170; p: 0.042*	103	r: 0.203; p: 0.040*	39	r: 0.041; p. 0.805
Phosphate	136	r: 0.067; p: 0.441	99	r: -0.054; p: 0.597	37	r: 0.353; p: 0.032*
alpha1-AR-AAB	141	r: -0.183; p: 0.030*	102	r: -0.286; p: 0.004*	38	r: 0.023; p. 0.889
M3-mAChR-AAB	144	r: -0.231; p: 0.005*	105	r: -0.303; p: 0.002*	38	r: -0.045; p: 0.787
Schellong examination:						
heart rate seated	132	r: -0.078; p: 0.373	95	r: 0.056; p: 0.589	36	r: -0.445; p: 0.007*
heart rate standing 0min	130	r: -0.200; p: 0.023*	94	r: -0.065; p: 0.535	35	r: -0.566; p: <0.001*
heart rate after 2min	129	r: -0.186; p: 0.035*	92	r: -0.063; p: 0.549	36	r: -0.433; p: 0.008*
heart rate after 5min	128	r. -0.197; p: 0.026*	91	r: -0.023; p: 0.826	36	r: -0.558; p: <0.001*
heart rate after 10min	82	r: -0.161; p: 0.148	62	r: -0.048; p: 0.713	19	r: -0.501; p: 0.025*
changes in heart rate from seated to standing	130	r: -0.231; p. 0.008*	94	r: -0.211; p: 0.041*	35	r: -0.203; p: 0.243
Clinical questionnaires:						
immune score	129	r: 0.141; p: 0.110	94	r: 0.241; p: 0.019*	34	r: -0.051; p: 0.776

*significant p <0.05.

In female patients with infection-triggered ME/CFS sCD26 correlated with the severity of immune-associated symptoms (r: 0.241; p: 0.019) and showed an inverse correlation with heart rate increase during orthostatic challenge (-0.211; p: 0.041). In patients without infection-triggered onset lower sCD26 concentrations were associated with POTS (p: 0.021). In line with this, sCD26 inversely correlated with heart rate in sitting (r: -0.445; p: 0.007) and standing posture after 0 (-0.566; p: <0.001), 2 (-0.433; p: 0.008), 5 (-0.558; p: <0.001) and 10 (-0.501; p: 0.025) minutes of orthostatic challenge.

We did not find sCD26 to correlate with patient’s symptoms after adjusting for multiple testing. In patients without infection-triggered onset inverse correlations of sCD26 concentrations with heart rate after 0 and 5 minutes of orthostatic challenge remained significant.

### Correlation of sCD26 With Immunological and Laboratory Parameters

Several correlations with immunological laboratory parameters were found in female infection-triggered ME/CFS. Soluble CD26 correlated with the percentage of CD8+ T cells expressing activation marker HLA-DR+ (r: 0.299; p: 0.005) and the number of CD4+CD8+ T cells (r: 0.270; p: 0.009), while inversely correlating with lymphocytes (r: -0.249; p: 0.017), CD3+ (r: -0.207; p: 0.048) and CD4+ (r: -0.221; p: 0.034) T cells and LPS-stimulated IL-1b (r: -0.309; p: 0.005). Furthermore, sCD26 correlated with levels of the enzymes lactate dehydrogenase (LDH) (r: 0.228; p: 0.020), creatin kinase (CK) (r: 0.329; p: 0.001), Glutamate-Pyruvate-Transaminase (GPT)/Alanine aminotransferase (ALT) (r: 0.271; p: 0.006), Glutamate-Oxaloacid-Transaminase (GOT)/Aspartate aminotransferase (AST) (r: 0.252; p: 0.010) and Gamma-Glutamyl-Transferase (GGT) (r: 0.203; p: 0.040). An inverse correlation was seen between sCD26 and levels of AAB against alpha1-AR (r: -0.286; p: 0.004) and M3-mAChR (r: -0.303; p: 0.002) (**Table 2A**).

In contrast, in patients without infection-triggered onset none of the above-mentioned correlations were seen and Spearman’s r even suggested an inverse relationship for some parameters. Soluble CD26 correlated only with phosphate (r: 0.353; p: 0.032) and inversely with Immunoglobulin A (IgA) levels (-0.366; p: 0.022) in this subgroup.

After applying BY-correction none of the correlations with laboratory parameters stayed significant. Interestingly, we observed similar correlation patterns in male patients (**Table 2B**, all data shown in **Supplementary Tables S3A–D**) as well as the whole ME/CFS cohort (**Table 2C**, all data shown in **Supplementary Tables S4A–C**). This particularly applies to inverse r-values for correlations between sCD26 and AAB. In the whole ME/CFS cohort most correlations with sCD26 stayed significant after BY-correction likely due to higher numbers (**Table 3**).

**Table 2B:** Parameters significantly correlating with sCD26 in male patients (onset stratified).

Parameter	Male ME/CFS cohort (n=60)		Male patients with infection-triggered onset (n=41)		Male patients without infection-triggered onset (n=18)	
	n	Spearman’s r; p	n	Spearman’s r; p	n	Spearman’s r; p
Laboratory assessment:						
ACE	47	r: -0.180; p: 0.225	32	r: -0.369; p: 0.038*	14	r: 0.209; p: 0.474
HLA-DR/CD8+ T-cells	50	r: 0.359; p: 0.010*	35	r: 0.377; p: 0.026*	14	r: 0.108; p: 0.713
LDH	58	r: 0.317; p: 0.015*	39	r: 0.369; p: 0.021*	18	r: 0.063; p: 0.804
HbA1c	55	r: 0.142; p: 0.301	37	r: 0.360; p: 0.029*	17	r: 0.065; p: 0.804
GPT	59	r: 0.354; p: 0.006*	40	r: 0.357; p: 0.024*	18	r: 0.327; p: 0.186
GOT	59	r: 0.367; p: 0.004*	40	r: 0.385; p: 0.014*	18	r: 0.265; p: 0.287
GGT	59	r: 0.259; p: 0.047*	40	r: 0.355; p: 0.024*	18	r: 0.044; p: 0.861
Potassium	57	r: -0.232; p: 0.079	39	r: -0.071; p: 0.663	17	r: -0.582; p: 0.014*
Thyroid peroxidase -AAB	35	r: -0.054; p: 0.759	22	r: 0.202; p: 0.368	13	r: -0.657; p: 0.020*
beta1-AR-AAB	59	r: -0.143; p: 0.280	40	r: -0.331; p: 0.037*	18	r: 0.273; p: 0.272
Schellong examination:						
heart rate seated	50	r: 0.314; p: 0.027*	34	r: 0.290; p: 0.096	15	r: 0.185; p: 0.510
heart rate after 5min	49	r: 0.251; p: 0.082	33	r: 0.368; p: 0.035*	15	r: -0.055; p: 0.845
RR (dia) seated	49	r: 0.280; p: 0.051	33	r: 0.348; p: 0.047*	15	r: 0.174; p: 0.536
RR (sys) standing 0min	49	r: 0.288; p. 0.045*	33	r: 0.306; p: 0.083	15	r: 0.267; p: 0.337
RR (dia) standing 0min	49	r: 0.370; p: 0.009*	33	r: 0.352; p: 0.045*	15	r: 0.262; p: 0.346
RR (sys) after 2min	49	r: 0.339; p: 0.017*	33	r: 0.354; p. 0.043*	15	r: 0.252; p: 0.364
RR (dia) after 2min	49	r: 0.445; p: 0.001*	33	r: 0.452; p: 0.008*	15	r: 0.259; p: 0.351
RR (sys) after 5min	49	r: 0.355; p: 0.012*	33	r: 0.406; p: 0.019*	15	r: 0.305; p. 0.269
RR (dia) after 5min	49	r: 0.382; p. 0.007*	33	r: 0.424; p: 0.014*	15	r: 0.282; p: 0.308
RR (sys) after 10min	33	r: 0.430; p. 0.012*	23	r: 0.463; p: 0.026*	9	r: -0.068; p: 0.862
RR (dia) after 10min	33	r: 0.612; p: <0.001*	23	r: 0.570; p: 0.004*	9	r: 0.528; p: 0.144
changes in heart rate from seated to standing	50	r: -0.281; p: 0.048*	34	r: -0.244; p: 0.165	15	r: -0.182; p: 0.515
changes in RR (sys) from seated to standing	49	r: 0.132; p. 0.365	33	r: 0.030; p: 0.868	15	r: 0.529; p: 0.043*
Clinical questionnaires:						
overall disease severity	51	r: -0.335; p: 0.016*	36	r: -0.360; p: 0.031*	14	r: -0.122; p: 0.679

*significant p <0.05.

**Table 2C:** Parameters significantly correlating with sCD26 in the whole ME/CFS cohort (onset stratified).

Parameter	Whole ME/CFS cohort (n=205)		Patients with infection-triggered onset (n=146)		Patients without infection-triggered onset (n=57)	
	n	Spearman’s r; p	n	Spearman’s r; p	n	Spearman’s r; p
Laboratory assessment:						
IL-1b	170	r: -0.182; p: 0.018*	118	r: -0.297; p: 0.001*	51	r: 0.107; p: 0.454
Ferritin	203	r: 0.144; p: 0.041*	144	r: 0.229; p: 0.006*	57	r: -0.056; p: 0.680
ACE	167	r: 0.121; p: 0.119	115	r: 0.041; p: 0.660	51	r: 0.307; p: 0.028*
lymphocytes	183	r: -0.168; p: 0.023*	129	r: -0.221; p: 0.012*	52	r: -0.065; p: 0.647
CD19+ B cells	182	r: 0.001; p: 0.986	129	r: -0.090; p: 0.312	52	r: 0.301; p: 0.030*
CD3+ T cells	183	r: -0.180; p: 0.015*	129	r: -0.215; p: 0.004*	52	r: -0.104; p: 0.464
CD4+ T cells	183	r: -0.207; p: 0.005*	129	r: -0.227; p: 0.010*	52	r: -0.196; p: 0.164
CD4+CD8+ T cells	183	r: 0.153; p: 0.039*	129	r: 0.139; p: 0.116	52	r: 0.208; p: 0.139
HLA-DR/CD8+ T-cells	172	r: 0.316; p: <0.001*	120	r: 0.345; p: <0.001*	51	r: 0.244; p: 0.085
IgA	203	r: <0.001; p: 0.996	144	r: 0.138; p: 0.098	57	r: -0.285; p: 0.032*
IgM	203	r: -0.093; p: 0.186	144	r: -0.179; p: 0.031*	57	r: 0.145; p: 0.282
LDH	201	r: 0.173; p: 0.014*	142	r: 0.272; p: 0.001*	57	r: -0.088; p: 0.510
CK	195	r: 0.258; p: <0.001*	139	r: 0.353; p: <0.001*	55	r: 0.051; p: 0.712
HbA1c	189	r: 0.114; p: 0.120	133	r: 0.183; p: 0.035*	55	r: -0.010; p: 0.943
GPT	202	r: 0.273; p: <0.001*	143	r: 0.349; p: <0.001*	57	r: 0.109; p: 0.418
GOT	202	r: 0.221; p: 0.002*	143	r. 0.336; p: <0.001*	57	r: -0.037; p: 0.787
GGT	202	r: 0.262; p: <0.001*	143	r: 0.326; p: <0.001*	57	r: 0.061; p: 0.650
Albumin	189	r: 0.121; p: 0.096	133	r: 0.197; p: 0.023*	55	r: -0.155; p: 0.259
Creatinine	205	r: 0.247; p: <0.001*	146	r: 0.252; p: 0.002*	57	r: 0.228; p: 0.089
Phosphate	193	r: -0.003; p: 0.968	138	r: -0.116; p: 0.175	54	r: 0.321; p: 0.018*
fT3	103	r: 0.206; p: 0.037*	68	r: 0.275; p: 0.023*	33	r: 0.007; p: 0.958
alpha1-AR-AAB	200	r: -0.172; p: 0.015*	142	r: -0.270; p: 0.001*	56	r: 0.067; p: 0.624
beta1-AR-AAB	202	r: -0.155; p: 0.027*	143	r: -0.201; p: 0.016*	57	r: 0.015; p: 0.913
beta2-AR-AAB	204	r: -0.111; p: 0.113	145	r: -0.198; p: 0.017*	57	r: 0.156; p: 0.245
M3-mAChR-AAB	203	r: -0.210; p: 0.003*	145	r: -0.304;p: <0.001*	56	r: 0.021; p: 0.879
Schellong examination:						
heart rate seated	182	r: -0.030; p: 0.685	129	r: 0.066; p: 0.459	51	r: -0.291; p: 0.038*
heart rate standing 0min	180	r: -0.148; p: 0.048*	128	r: -0.041; p: 0.646	50	r: -0.438; p: 0.001*
heart rate after 2min	179	r: -0.103; p: 0.171	126	r: 0.001; p: 0.993	51	r: -0.366; p: 0.008*
heart rate after 5min	177	r: -0.106; p: 0.162	124	r: 0.053; p: 0.559	51	r: -0.450; p: 0.001*
heart rate after 10min	115	r: -0.126; p: 0.181	85	r: -0.015; p: 0.894	29	r: -0.420; p: 0.023*
RR (sys) seated	180	r: 0.159; p: 0.034*	127	r: 0.204; p: 0.022*	51	r: 0.052; p: 0.717
RR (sys) standing 0min	178	r: 0.185; p: 0.014*	126	r: 0.209; p: 0.019*	50	r: 0.181; p: 0.208
RR (sys) after 2min	176	r: 0.210; p: 0.005*	123	r: 0.272; p: 0.002*	51	r: 0.091; p: 0.525
RR (sys) after 5min	177	r: 0.225; p: 0.003*	124	r: 0.282; p: 0.002*	51	r: 0.119; p: 0.404
RR (dia) after 5min	177	r: 0.189; p: 0.012*	124	r: 0.245; p: 0.006*	51	r: 0.059; p: 0.679
changes in heart rate from seated to standing	180	r: -0.226; p: 0.002*	128	r: -0.203; p: 0.022*	50	r: -0.256; p: 0.073
changes in RR (sys) from seated to standing	178	r: 0.073; p: 0.333	126	r: -0.009; p: 0.920	50	r: 0.289; p: 0.042*
Clinical questionnaires:						
immune score	187	r: 0.115; p: 0.116	133	r: 0.197; p: 0.023*	52	r: -0.022; p: 0.878

*significant p <0.05.

**Table 3:** Parameters significantly correlating with sCD26 in the whole ME/CFS cohort after BY-correction (onset stratified).

Parameter	Whole ME/CFS cohort (n=205)		Patients with infection-triggered onset (n=146)		Patients without infection-triggered onset (n=57)	
	n	Spearman’s r;BY-corrected p	n	Spearman’s r;BY-corrected p	n	Spearman’s r;BY-corrected p
Laboratory assessment:						
IL-1b	170	r: -0.182; p: 0.396	118	r: -0.297; p: 0.035*	51	r: 0.107; p: >0.999
HLA-DR/CD8+ T-cells	172	r: 0.316; p: 0.007*	120	r: 0.345; p: 0.006*	51	r: 0.244; p: >0.999
LDH	201	r: 0.173; p: 0.366	142	r: 0.272; p: 0.035*	57	r: -0.088; p: >0.999
CK	195	r: 0.258; p: 0.019*	139	r: 0.353; p: 0.003*	55	r: 0.051; p: >0.999
GPT	202	r: 0.273; p: 0.012*	143	r: 0.349; p: 0.003*	57	r: 0.109; p: >0.999
GOT	202	r: 0.221; p: 0.072	143	r. 0.336; p: 0.004*	57	r: -0.037; p: >0.999
GGT	202	r: 0.262; p: 0.015*	143	r: 0.326; p: 0.005*	57	r: 0.061; p: >0.999
Creatinine	205	r: 0.247; p: 0.019*	146	r: 0.252; p: 0.059	57	r: 0.228; p: >0.999
alpha1-AR-AAB	200	r: -0.172; p: 0.366	142	r: -0.270; p: 0.035*	56	r: 0.067; p: >0.999
M3-mAChR-AAB	203	r: -0.210; p: 0.100	145	r: -0.304; p: 0.009*	56	r: 0.021; p: >0.999
Schellong examination:						
heart rate standing 0min	180	r: -0.148; p: 0.428	128	r: -0.041; p: >0.999	50	r: -0.438; p: 0.046*
heart rate after 5min	177	r: -0.106; p: 0.875	124	r: 0.053; p: >0.999	51	r: -0.450; p: 0.046*

* significant p <0.05; all p-values BY-corrected.

**Figure 3:**
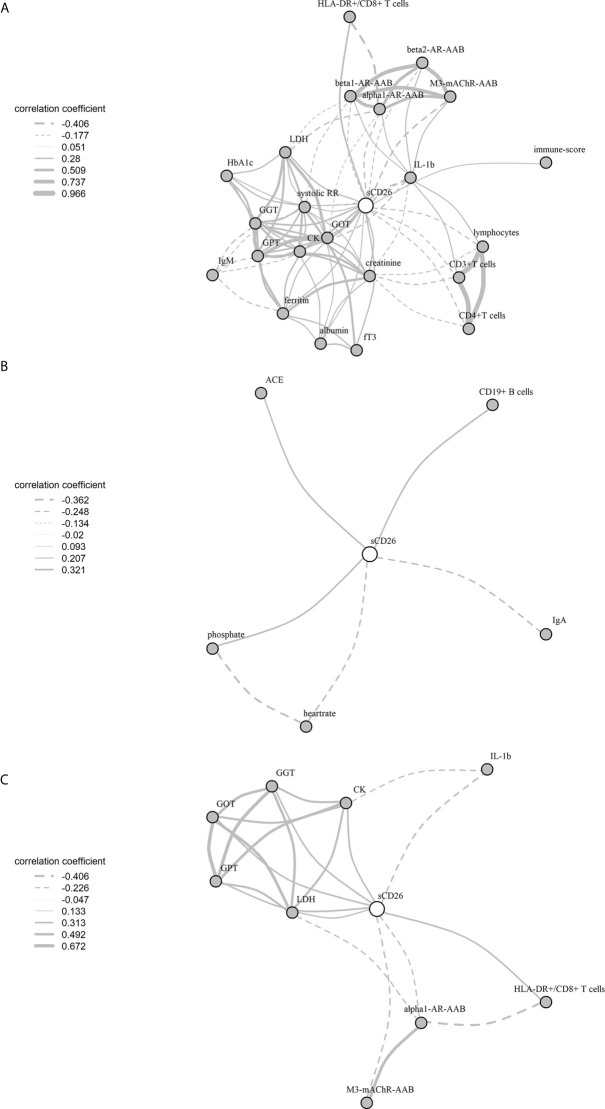
Network of correlations with sCD26 in ME/CFS. Fruchterman-Reingold-algorithm-based network graph for correlations with sCD26 in patients with infection-triggered onset **(A)** (n=146), patients without infection-triggered onset **(B)** (n=57) as well as patients with infection-triggered onset after BY-correction **(C)**. Each edge (line) of the graph represents a spearman correlation with p<0.05 between two parameters. The width of the edge represents the absolute nonparametric spearman correlation coefficient r. Solid lines depict positive correlations, dashed lines negative correlations.

Significant correlations between sCD26 and laboratory parameters and their intercorrelations were visualized in a network graph (**Figure 3**). In patients with infection-triggered onset we observed strong intercorrelations between markers of organ function and damage GPT, GOT, GGT, LDH and CK as well as AAB. Further, analyses revealed inverse correlations of HLA-DR+ expressing CD8+ T cells and LDH with alpha1-AR-AAB. CK correlated inversely with IL-1b.

### Regression Analyses for sCD26

**Figure 4:**
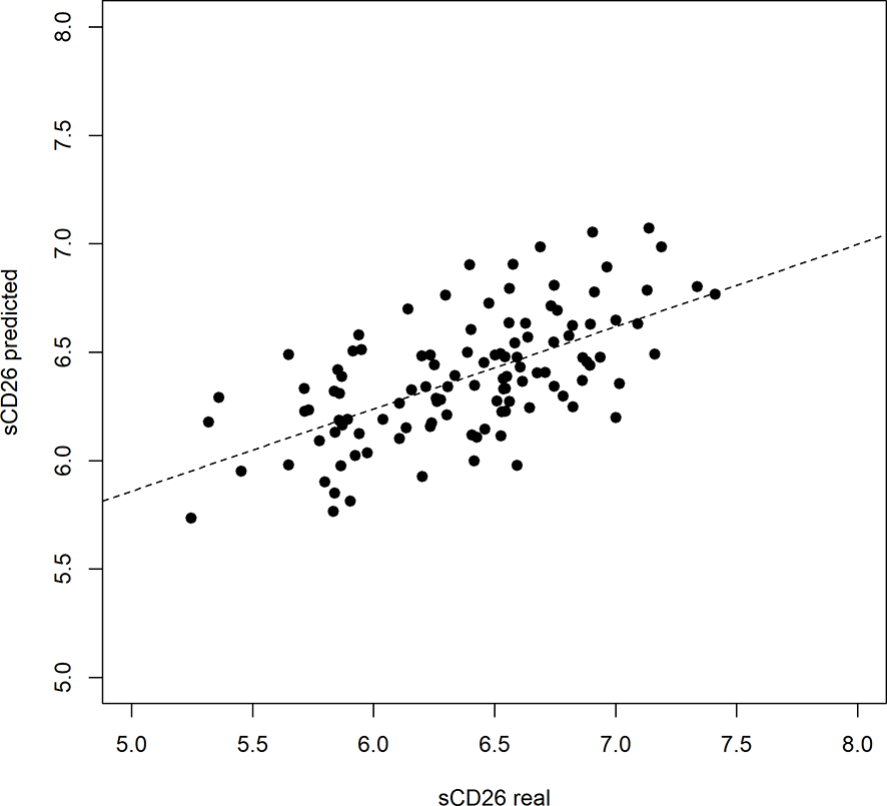
Performance of regression model on sCD26 log(sCD26) values predicted by regression model plotted against real log(sCD26) values. Dashed diagonal line represents estimated fit.

Based on significant correlations after BY-correction (**Table 3**) we performed a multiple regression analysis for sCD26 in order to quantify the strength of the associations. Since correlation analyses showed considerable differences between onset groups, we focused on patients with infection-triggered ME/CFS. Sex and age were included as possible confounders. The model was significant (p: <0.001) with an adjusted R^2^ of 0.325. Bi-directional stepwise regression resulted in the best fitting model (adjusted R^2^: 0.344; p: <0.001) consisting of the parameters shown in **Table 4**. In **Figure 4** predicted log(sCD26) values are plotted against real ones (consult **Supplementary Figures S1A–C**) for additional diagnostic plots concerning this linear regression analysis).

**Table 4:** Model-coefficients after stepwise multiple regression on sCD26 in infection-triggered ME/CFS.

	Estimate	Standard Error	t value	p
(Intercept)	6.569	0.572	11.484	<0.001*
GPT	0.246	0.089	2.757	0.007*
GGT	0.123	0.081	1.511	0.134
HLA-DR+/CD8+ T cells	0.221	0.047	4.757	<0.001*
M3-mAChR-AAB	-0.166	0.084	-1.977	0.051
IL-1b	-0.120	0.052	-2.295	0.024*
age	-0.251	0.123	-2.040	0.044*

*significant p <0.05.

### CD26 Expression on Immune Cells

**Figure 5:**
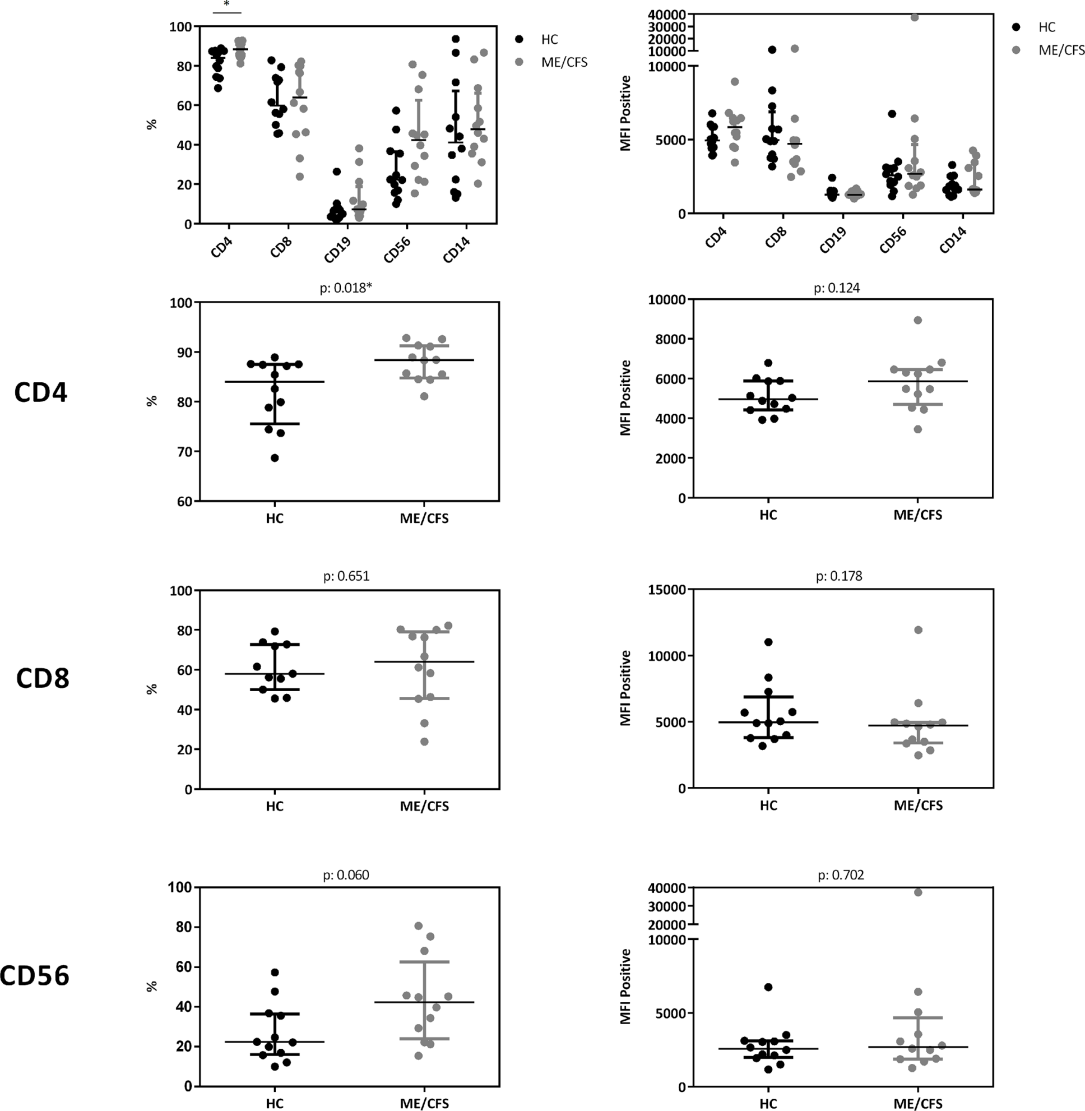
CD26 expression on immune cells. Comparison of CD26 surface expression on immune cells as percentage of expressing cells (left) as well as expression level per cell (MFI) (right) from flow cytometry analyses. Percentage of CD26+ expressing cells and MFI between ME/CFS and HC were compared using Mann-Whitney-U rank-sum test (*p < 0.05).

In a cohort of HC (n: 12) and ME/CFS (n: 12) patients we analyzed immune cells for their CD26 surface expression. Median age of ME/CFS patients analyzed was 53 years (IQR: 33-57), median age of HC 34 years (IQR: 29-42). All subjects analyzed were female. We observed significantly increased frequencies of CD26 expressing CD4+ T cells (p: 0.018), but no difference in the expression level of CD26 (Median Fluorescence Intensity (MFI)) per cell (**Figure 5**, gating strategy in **Supplementary Figure S2**).

## Discussion

In this study we re-valuated sCD26 in the context of ME/CFS. Though we could not confirm its diagnostic suitability, results from correlation analyses provide some striking pathomechanistic insights.

Our study is partially in accordance with a previous study comparing 73 patients with ME/CFS with 122 HC showing diminished concentrations of sCD26 (23). We found lower concentrations of sCD26 only in female ME/CFS patients as compared to female HC. ROC analysis in our study could not replicate diagnostic suitability of sCD26. Further, in line with our findings of increased frequencies of CD26 expressing CD4+ T cells, Fletcher *et al* found an elevated number of T and NK cells expressing CD26 in ME/CFS. The CD26 expression level per cell was not enhanced in ME/CFS in our study and Fletcher *et al* even found a decrease in CD26 expression level on T cells and NK cells. The underlying mechanism of reduced sCD26 in various autoimmune diseases is still not resolved. A recent study showed that sCD26/DPP-4 is stored in T cells in secretory granules together with granzymes and perforin. Upon stimulation degranulation leads to a massive release of proteolytically active sCD26/DPP-4 (16). In line with this, serum concentration of sCD26 were shown to depend on the activation state of T cells (15).

Further, we found higher levels of sCD26 in male patients with postinfectious onset compared to noninfectious onset. In a previous study, Porter *et al.* provided evidence for a different regulation of CD26 among onset subsets as they found higher numbers of CD26+CD4+ T cells in postviral vs. non-viral onset ME/CFS patients (24). As we analyzed CD26 expression only in a small subgroup we could not compare subgroups.

When performing correlation analyses, we observed that concentrations of sCD26 are associated with immunological and cardiovascular parameters as well as liver enzymes and CK in ME/CFS. Remarkably, most associations were only observed in patients with infection-triggered disease. Of the correlations that stayed significant after BY-correction, one of the most striking findings of our study is the inverse correlation of alpha1-AR- and M3-mAChR-AAB with sCD26 in infection-triggered ME/CFS, but not in patients with non-infection-triggered onset. We and others found elevated beta1/2-AR- and M3-mAChR-AAB in a subset of ME/CFS patients (6). Higher levels of autoantibodies to alpha1-AR and M3-mAChR were found in patients with both orthostatic hypotension and POTS, too (32, 33). In our study we observed an inverse correlation between alpha1-AR-AAB and percentage of HLA-DR+CD8+ T-cells. It was shown that T cells also express alpha1-AR which mediates inhibition of T cell proliferation (34). Thus, it is tempting to speculate that higher alpha1-AR-AAB are associated with inhibition of activation of T cells although it is not known if alpha1-AR-AAB can activate the receptor. In line with the finding that activated T cells are a major source of sCD26 (15, 16) it is plausible that lower percentages of HLA-DR+ in CD8+ T-cells are directly associated with lower sCD26.

IL-1 release was found to be associated inversely with sCD26. A direct enzymatical degradation of IL-1 by sCD26 remains controversial (35). We could observe in a recent study beta2-AR-AAB to inhibit LPS-induced tumor necrosis factor (TNF) release, and their inhibitory function was impaired in ME/CFS with higher beta2-AR-AAB (36). This may be an explanation for an association of beta2-AR-AAB with IL-1 release observed before BY-correction in patients with infection-triggered onset. However, we found no correlation of sCD26 with TNF release, which may be related to the fact that these cytokines are differentially regulated in monocytes and macrophages.

Further, we found a strong association of GOT, GPT and GGT with sCD26 in line with the well-known association of DPP-4 with liver and adipose tissue inflammation and its expression on bile ducts (37–40). As CD26 plays an important role in blood glucose level regulation by cleaving Glucagon-like-peptide 1 (GLP-1) (41), CD26/DPP-4 inhibitors are widely used in the treatment of diabetes mellitus (42). In line with our findings, DPP-4 inhibitors improve liver dysfunction in type 2 diabetes mellitus (43). Correlations between sCD26 and CK match reports of sCD26 being shed from muscles as a myokine (44). In line with findings by Nacul *et al.* low sCD26 levels in ME/CFS might to some extent be linked to physical inactivity and impaired muscle energy metabolism associated with PEM (45). A correlation of sCD26 with LDH could be explained through its release by liver cells as well as the above discussed reduction of oxidative stress observed as a result of DPP-4 inhibition.

We observed correlations of sCD26 concentrations with blood pressure in patients with infection-triggered onset, which, however, did not remain after BY-correction. Remarkably, in patients with non-infection-triggered onset the inverse correlation of sCD26 with heart rate upon orthostatic challenge remained. Patients from this group who suffered from POTS had significantly lower sCD26 concentrations, too. Low blood volume has been described in POTS and linked to disturbance in renin-angiotensin-aldosterone system (RAAS) (46, 47). We found a correlation of sCD26 with ACE in this group before BY-correction. The observed associations in this group may point to an impaired orthostatic regulation enhancing preexisting disturbances caused by POTS.

As 95% of the serum DPP-4 activity origins from sCD26 we tried to provide a mechanistic explanation of the association of sCD26 with immunological, cardiovascular and liver parameters in patients based on the known effect of DPP-4 inhibition observed in various studies (14). A hypothesized model of the regulation and role of sCD26 in patients with ME/CFS is depicted in the **Supplementary Figure S3**.

## Limitations and Strengths

Our study has several limitations. The ME/CFS cohort and the HC group were not well matched for age and sex, but this was considered in our analyses. Many associations we discuss are based on the effect of DPP-4 inhibitors. We did not analyze the DPP-4 activity of sCD26. However, Durinx *et al.* showed, that serum dipeptidyl peptidase activity origins mostly from sCD26 (14). In SLE concentrations of sCD26 closely correlated with DPP-4 activity (48). Further, we do not know the contribution of cellular CD26 to the overall DPP-4 activity. Our concept has to be considered hypothetical and needs confirmation in further patient cohorts and experimental studies.

The strength of our study is that we provide evidence that the key enzyme sCD26 is linked to immunological and cardiovascular disturbances in ME/CFS. The associations of sCD26 with autoantibodies provide further evidence for the autoimmune pathomechanism of infection-triggered ME/CFS and the delineation of ME/CFS subgroups (12). These findings may help to elucidate heterogeneity of patient’s symptoms and biomarkers found in most studies on ME/CFS (49).

## Data Availability Statement

The original contributions presented in the study are included in the article/**Supplementary Material**. Further inquiries can be directed to the corresponding author.

## Ethics Statement

The studies involving human participants were reviewed and approved by Ethics Committee of Charité Universitätsmedizin Berlin (EA2/038/14 and EA2/067/20). The patients/participants provided their written informed consent to participate in this study.

## Author Contributions

MS, HF, FS, and JH performed the research. MS, SL, and NS participated in data analysis. CK, PG, KW, and LH contributed with patient material. CS designed the research project. MS and CS wrote the paper. All authors contributed to the article and approved the submitted version.

## Funding

This work was supported by a grant from the Weidenhammer-Zöbele Foundation. MS received a scholarship from the Lost Voices Foundation. We acknowledge support from the German Research Foundation (DFG) and the Open Access Publication Fund of Charité–Universitätsmedizin Berlin.

## Conflict of Interest

Author HH was employed by CellTrend GmbH. CellTrend GmbH holds a patent on the use of beta-adrenergic receptor antibodies in diagnosis of ME/CFS. CS has a consulting agreement with Celltrend.

The remaining authors declare that the research was conducted in the absence of any commercial or financial relationships that could be construed as a potential conflict of interest.​

## Supplementary Material

Supplementary Figure S1(A), S1(B), S1(C) Residual plot, Q-Q plot and Cook’s distance for multiple regression.

Supplementary Figure S2 MFI-gating strategy.

Supplementary Figure S3 Hypothesized model of the regulation and role of sCD26 in ME/CFS.

Supplementary Table S1 Characteristics and co-morbidities.

Supplementary Table S2(A-D) Correlations of sCD26 with cohort characteristics, laboratory assessments, Schellong examination results and clinical questionnaire outcome in female patients.

Supplementary Table S3(A-D) Correlations of sCD26 with cohort characteristics, laboratory assessments, Schellong examination results and clinical questionnaire outcome in male patients.

Supplementary Table S4(A-C) Correlations of sCD26 with laboratory assessments, Schellong examination results and clinical questionnaire outcome in all patients.

Supplementary Data Sheet 1 Assessment of ME/CFS patients (age, sex, comorbidities, sCD26 concentrations, laboratory and symptom assessment, results from orthostatic challenge).

Supplementary Data Sheet 2 Assessment of HC (age, sex, sCD26 concentrations).

Supplementary Data Sheet 3 Assessment of sCD26 expression on immune cells in ME/CFS and HC (percentages of CD26 expressing cells, MFI).

## Abbreviations

AAB, Autoantibodies; ACE, Angiotensin converting enzyme; Alpha1/2-AR, Alpha 1/2 adrenergic receptor; ALT, Alanine aminotransferase, also known as Glutamate-Pyruvate-Transaminase (GPT); ANCA-associated vasculitides, Antineutrophil cytoplasmatic antibody-associated vasculitides; AST, Aspartate aminotransferase, also known as Glutamate-Oxaloacid-Transaminase (GOT); AT1-R, Angiotensin-II-receptor type 1; Beta1/2/3-AR, Beta 1/2/3 adrenergic receptor; BY, Benjamini-Yekutieli; CD26, cluster of differentiation 26; CI, Confidence interval; CK, Creatin kinase; COMPASS 31, Composite Autonomic Symptom Score 31; CTLA4, Cytotoxic T lymphocyte associated protein 4; DPP-4, Dipeptidyl peptidase 4; EBV, Epstein-Barr virus; ETA-R/ETB-R, Endothelin receptor A/B; fT3, Free triiodothyronine; GGT – Gamma-Glutamyl-Transferase; GLP-1, Glucagon-like-peptide 1; GOT, Glutamate-Oxaloacid-Transaminase, also known as Aspartate aminotransferase (AST); GPT, Glutamate-Pyruvate-Transaminase, also known as Alanine aminotransferase (ALT); GPCR, G-protein coupled receptor; HbA1c, Glycated hemoglobin; HC, Healthy controls; IBS, Irritable bowel syndrome; IgA, Immunoglobulin A; IgM, Immunoglobulin M; IL-1b, Interleukin-1 beta; IQR, Interquartile range; LPS, Lipopolysaccharide; LDH, Lactate dehydrogenase; M3/4-mAChR, Muscarinic acetylcholine receptor ¾; ME/CFS, Myalgic Encephalomyelitis/Chronic fatigue syndrome; MFI, Median Fluorescence Intensity; n, Number; PBMC, Peripheral blood mononuclear cell; POTS, Postural orthostatic tachycardia syndrome; PTPN22, Protein tyrosine phosphatase non-receptor type 22; RAAS, Renin-angiotensin-aldosterone system; ROC, Receiver operating characteristic; SD, Standard deviation; SF-36, Short Form Health Survey 36; TNF, Tumor necrosis factor.
