# Supplementary material for: Delineating the Association Between Soluble CD26 and Autoantibodies Against G-Protein Coupled Receptors, Immunological and Cardiovascular Parameters Identifies Distinct Patterns in Post-Infectious vs. Non-Infection-Triggered Myalgic Encephalomyelitis/Chronic Fatigue Syndrome
Source: Front Immunol. 2021 Apr 6;12:644548. doi: 10.3389/fimmu.2021.644548 (PMC8056217; doi:10.3389/fimmu.2021.644548)
Supplement: Supplementary Figure S1(A), S1(B), S1(C) — Residual plot, Q-Q plot and Cook’s distance for multiple regression. [file DataSheet_4.docx]

Supplementary Material

# Supplementary Figures

## Figures S1(A)-(C): Supplements for stepwise regression analyses

### Figure S1(A): Residual plot


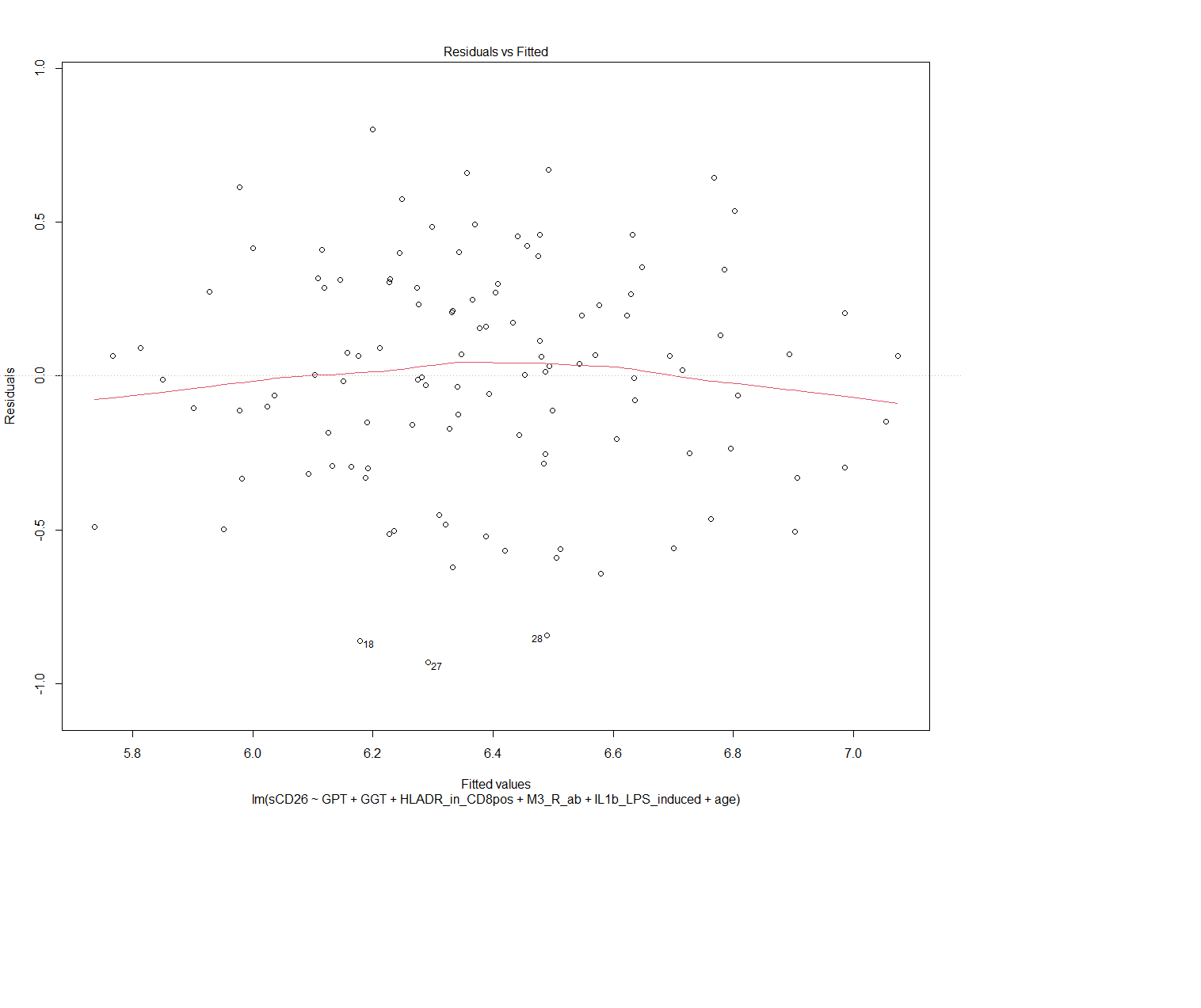


Figure S1(A): Residuals were plotted against fitted values. The plot serves to understand whether the residuals have the same spread across all predicted values (homoscedasticity of residuals). This criterion was fulfilled for the performed regression analysis.

### Figure S1(B): Q-Q plot


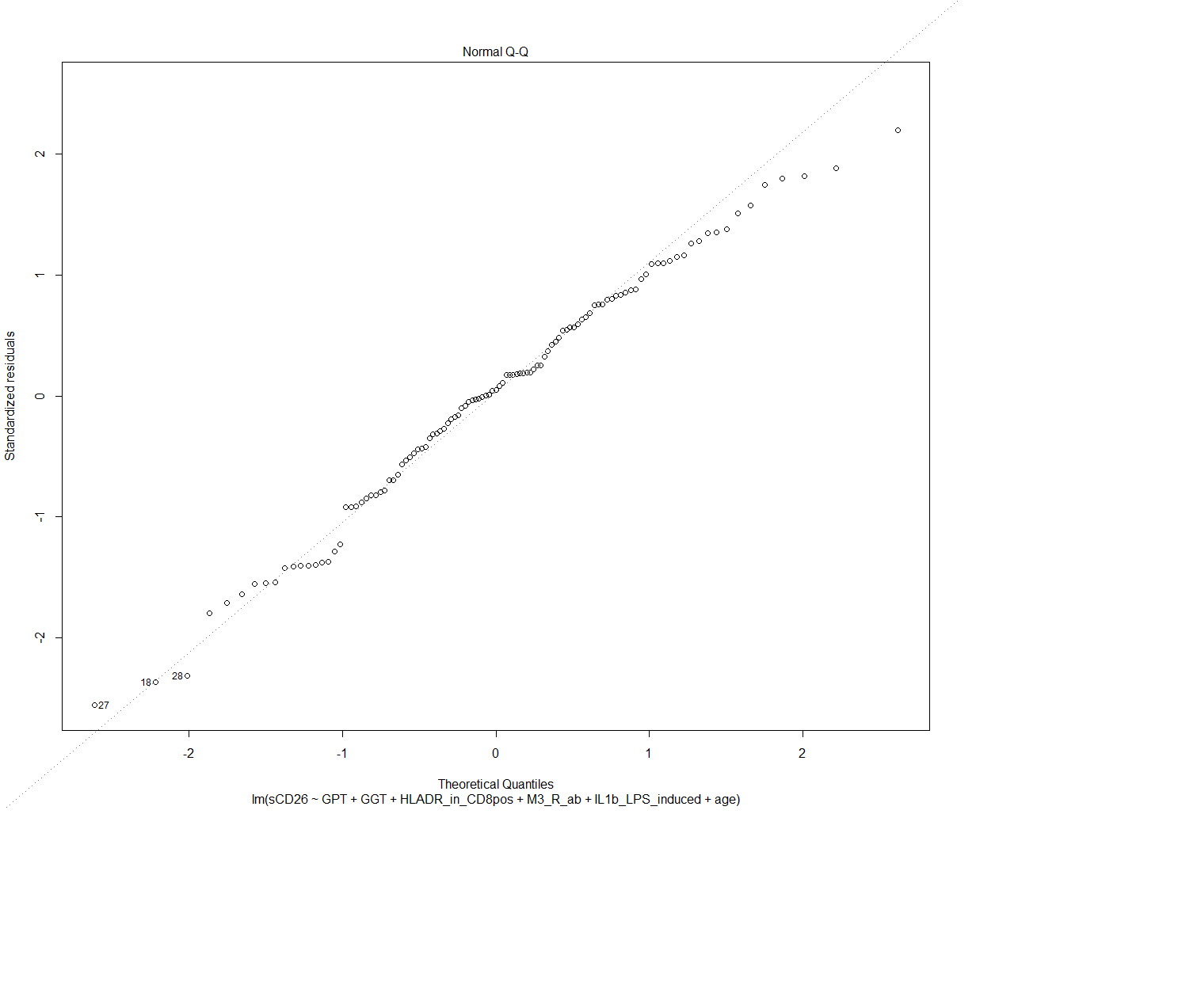


Figure S1(B): The Q-Q-plot assesses the normal distribution of the residuals. The dashed line represents a perfect normal distribution. Residuals of our regression analysis meet this criterion.

### Figure S1(C): Cook’s distance plot


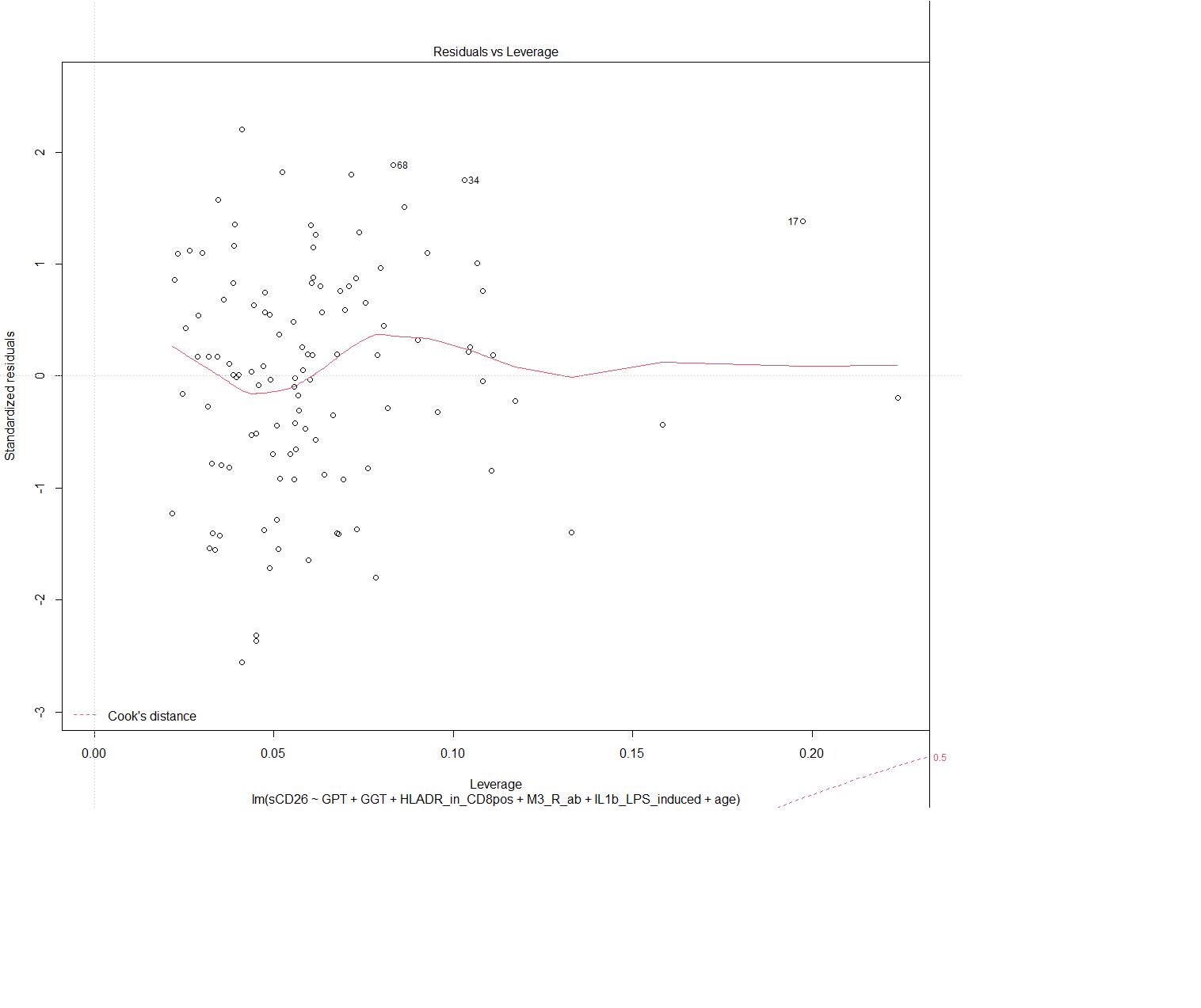


Figure S1(C): The Cook’s distance plot evaluates the effect of each single observation in the model fitting. In this case no extreme values were detected.

## Figure S2: Gating strategy for CD26 expression on immune cells


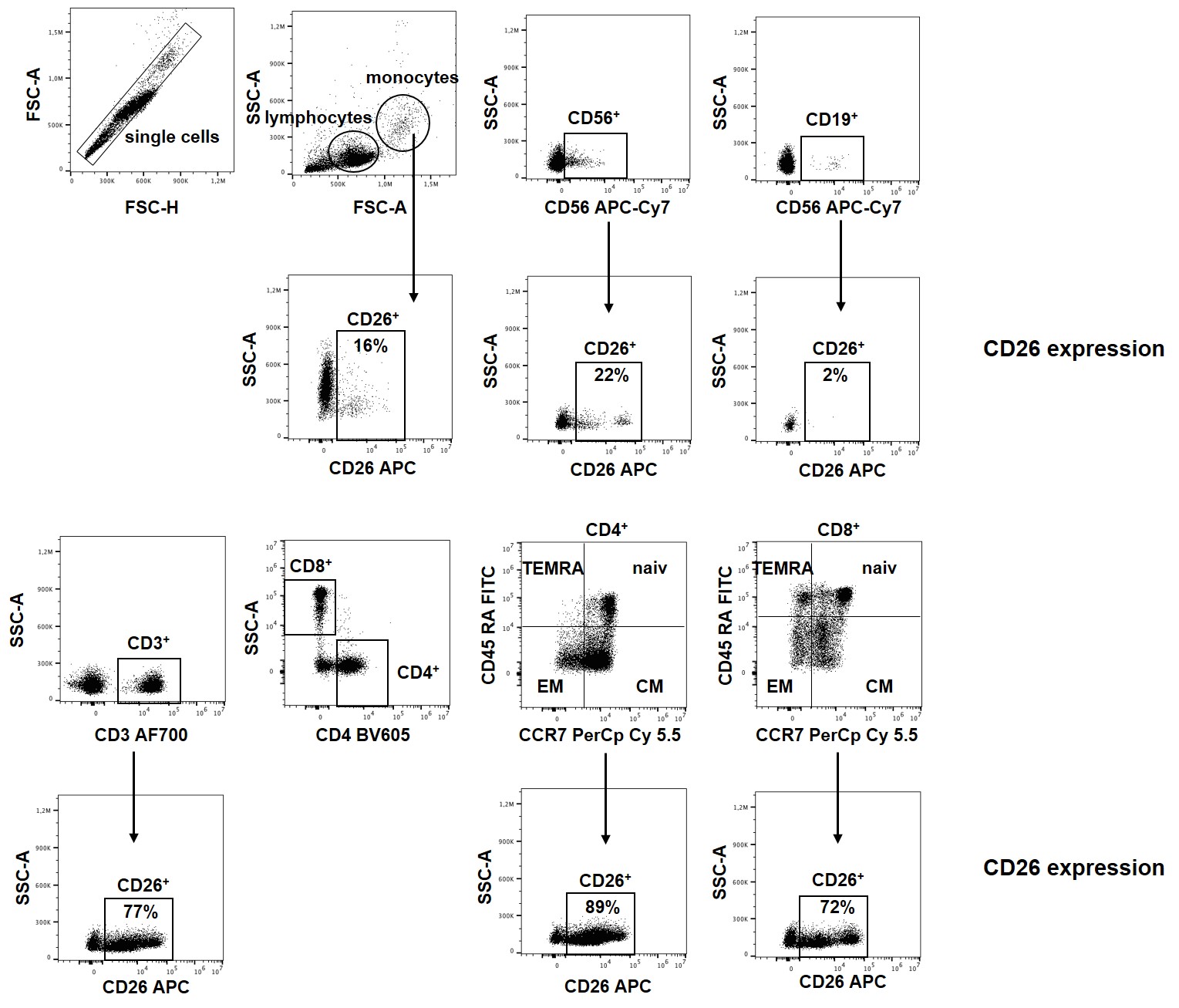


## Figure S3: Hypothesized model of the regulation and role of sCD26 in ME/CFS


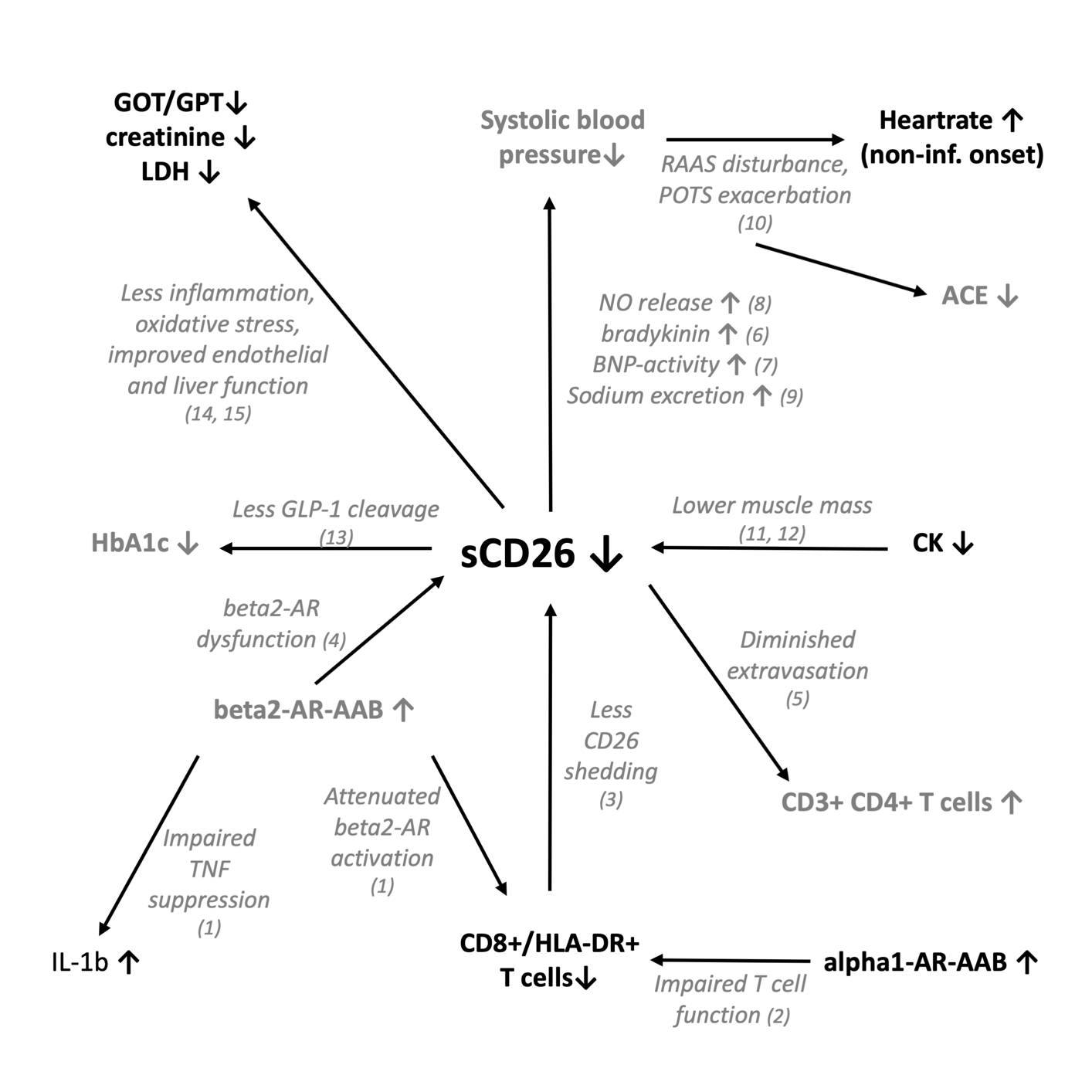


Significant correlations after BY-correction are depicted in black, correlations only significant before BY-correction in grey. Evidence for suggested mechanisms is noted along the arrows. AAB against alpha1-AR and beta2-AR mediate adrenergic dysfunction resulting in reduced sCD26 production, impaired T cell activation and enhanced IL-1b production (1-4). Lower concentrations of sCD26 are likely to lead to impaired lymphocyte migration and extravasation (5). Lower sCD26 is associated with lower systolic blood pressure via higher BNP and bradykinin activity, higher NO-release and enhanced sodium excretion (6-9). In patients without infection-triggered onset lower sCD26 leads to increased heart rate after orthostatic challenge, underlying POTS might exacerbate via RAAS-paradoxon (10). Low sCD26 correlates with low CK due to less muscular shedding and lower muscle mass in ME/CFS (11, 12). In line with the therapeutic target of DPP-4 inhibitors lower sCD26 correlates with lower HbA1c (13). Correlations of low sCD26 were seen with improved liver function and decreased creatinine (14, 15).

# Supplementary Tables

**Table S1**: Characteristics and co-morbidities

| parameter | | Whole cohort (n: 205) | | | Patients with infection-triggered onset (n: 146) | | | Patients with non-infection-triggered onset (n: 57) | | | inf vs. non-inf onset |
| --- | --- | --- | --- | --- | --- | --- | --- | --- | --- | --- | --- |
|  |  | median (IQR)/% | n (criteria positive) | sCD26 Correlation/  Mann-U results | median (IQR)/% | n (criteria positive) | sCD26 Correlation/  Mann-U results | median (IQR)/% | n (criteria positive) | sCD26 Correlation/  Mann-U results |  |
| age | a | 43 (32.5-50.5) | 205 | r: 0.081; p: 0.249 | 40 (31-48) | 146 | r: 0.094; p: 0.261 | 49 (42-54) | 57 | r: 0.255; p. 0.056 | p <0.001 |
| sex |  | 71% female | 205 (145f/60m) | **p: 0.007*** (f<m) | 72% female | 146 (105f/41m) | **p: 0.002*** (f<m) | 68% female | 57 (39f/18m) | p: 0.945 |  |
| Infection-triggered disease onset |  | 72% | 203 (146) | p: 0.525 | - | - | - | - | - | - |  |
| onset trigger Ebstein-Barr virus |  | 17% | 200 (33) | p: 0.522 | 23% | 142 (33) | p: 0.416 | - | - | - |  |
| disease duration | a | 4 (2-9) | 205 | r: -0.025; p: 0.720 | 3 (2-8) | 146 | r: -0.097; p: 0.244 | 5 (2-12.5) | 57 | r: 0.124; p: 0.360 |  |
| Comorbidities:  Recurrent respiratory tract infections  Herpes  Allergies  Irritable bowel symptoms  Food intolerances  Depression  Fibromyalgia  Hashimoto  POTS |  | 56%  52%  43%  66%  34%  27%  10%  11%  16% | 204 (114)  203 (105)  204 (88)  204 (135)  204 (70)  204 (56)  205 (21)  204 (23)  179 (29) | p: 0.176  p: 0.601  p: 0.213  p: 0.795  p: 0.600  p: 0.294  p: 0.465  p: 0.276  p: 0.258 | 66%  49%  44%  66%  39%  24%  6%  12%  18% | 145 (96)  145 (71)  145 (64)  145 (96)  145 (57)  145 (35)  146 (9)  145 (17)  126 (23) | p: 0.349  p: 0.968  p: 0.905  p: 0.353  p: 0.818  p: 0.432  p: 0.964  p: 0.854  p: 0.972 | 30%  57%  42%  67%  23%  33%  19%  11%  12% | 57 (17)  56 (32)  57 (24)  57 (38)  57 (13)  57 (19)  57 (11)  57 (6)  51 (6) | p: 0.402  p: 0.389  p: 0.084  p: 0.352  p: 0.351  p: 0.229  p: 0.175  p: 0.089  **p: 0.009*** (sCD26 lower in POTS) | p <0.001  p 0.027  p 0.005 |

**Table S2(A)**: Correlations between sCD26 and group characteristics in female ME/CFS patients (onset stratified)

| parameter | | Female patients (n=145) | | | Female patients with infection triggered onset (n=105) | | | Female patients with non-infection-triggered onset (n=39) | | |
| --- | --- | --- | --- | --- | --- | --- | --- | --- | --- | --- |
|  |  | median (IQR)/% | n (criteria pos) | Correlation/Mann-U | median (IQR)/% | n (criteria pos) | Correlation/Mann-U | median (IQR)/% | n (criteria pos) | Correlation/Mann-U |
| age | a | 44 (33-51) | 145 | **r: 0.175; p: 0.035*** | 40 (31.5-49) | 105 | r: 0.144; p: 0.141 | 49 (44-53) | 39 | **r: 0.441; p: 0.009*** |
| Infection-triggered disease onset |  | 73% | 144 (105) | p: 0.755 | - | - | - | - | - | - |
| onset trigger Ebstein-Barr virus |  | 19% | 140 (26) | p: 0.265 | 26% | 101 (26) | p: 0.277 | - |  | - |
| disease duration | a | 4 (2-9,5) | 145 | r: 0.034; p 0.689 | 3 (1-8.5) | 105 | r: -0.047; p: 0.634 | 6 (2-14) | 39 | r: 0.141; p: 0.393 |

**Table S2(B)**: Correlations between sCD26 and laboratory assessments in female ME/CFS patients (onset stratified)

| parameter | | Female patients (n=145) | | | | Female patients with infection triggered onset (n=105) | | | | Female patients with non-infection-triggered onset (n=39) | | | |
| --- | --- | --- | --- | --- | --- | --- | --- | --- | --- | --- | --- | --- | --- |
|  |  | median (IQR)/% | n (criteria pos) | Correlation/Mann-U | BY-corrected p | median (IQR)/% | n (criteria pos) | Correlation/Mann-U | BY-corrected p | median (IQR)/% | n (criteria pos) | Correlation/Mann-U | BY-corrected p |
| Immunological assessment:  CrP  IL-1b  soluble IL-2 receptor  Ferritin  Angiotensin converting enzyme  C3 complement  C4 complement  lymphocytes  monocytes  granulocytes  natural killer (NK) cells  CD19+ B cells  CD3+ T cells  CD4+ T cells  CD8+ T cells  CD4+CD8+ T cells  CD4/CD8-ratio  HLA-DR+/CD8+ T cells  CD11a+/CD8- T cells  CD28+/CD8+ T cells  CD57+/CD8- T cells  monocytal HLA-DR expr.  ANA >1:160  IgG  IgA  IgM  IgE | mg/dl  pg/ml  IU/ml  µg/ml  U/l  mg/dl  mg/dl  /nl  /nl  /nl  /nl  /nl  /nl  /nl  /nl  %  %  %  %  %  ab/cell  g/l  g/l  g/l  kU/l | 1.50 (0.60-3.00)  224.00 (149-409)  332.00 (262-435)  53.00 (30-86)  26.70 (20-33)  1020 (880-1200)  230 (170-270)  1.93 (1.55-2.33)  0.41 (0.34-0.56)  4.20 (3.18-5.26)  0.17 (0.13-0.27)  0.22 (0.16-0.28)  1.43 (1.15-1.79)  0.93 (0.77-1.17)  0.40 (0.31-0.54)  1.22 (0.79-1.99)  2.30 (1.70-2.90)  11 (6-19)  52 (39-66)  80 (69-88)  2 (1-4)  40819 (33731-48626)  14%  9.76 (8.40-11.09)  1.61 (1.21-2.03)  1.16 (0.75-1.58)  23.30 (10.75-80.00) | 143  120  140  143  120  123  123  131  131  131  131  130  131  131  131  131  131  122  120  122  122  129  120 (17)  143  143  143  137 | r: 0.011; p: 0.893  r: -0.171; p: 0.062  r: 0.060; p: 0.484  r: 0.039; p: 0.645  **r: 0.218; p: 0.017***  r: -0.029; p: 0.750  r: -0.048; p: 0.596  **r: -0.218; p: 0.013***  **r: -0.181; p: 0.039***  r: -0.097; p: 0.272  r: -0.140; p: 0.110  r: -0.040; p: 0.655  **r: -0.214; p: 0.014***  **r: -0.231; p: 0.008***  r: -0.084; p: 0.339  **r: 0.230; p: 0.008***  r: -0.139; p: 0.114  **r: 0.312; p: <0.001***  **r: 0.221; p: 0.015***  r: -0.162; p: 0.075  r: 0.085; p: 0.351  r: 0.089; p: 0.315  p: 0.961  r: -0.124; p: 0.140  r: -0.112; p: 0.181  r: -0.031; p: 0.713  r: -0.115; p: 0.179 | >0.999  >0.999  >0.999  >0.999  0.489  >0.999  >0.999  0.489  0.870  >0.999  >0.999  >0.999  0.489  0.436  >0.999  0.436  >0.999  0.125  0.489  >0.999  >0.999  >0.999  >0.999  >0.999  >0.999  >0.999 | 1.80 (0.60-3.00)  210.00 (137-409)  330.00 (264-396)  47.60 (24-76)  25.40 (20-32)  995 (878-1183)  230 (170-270)  1.93 (1.54-2.41)  0.41 (0.34-0.56)  4.25 (3.22-5.26)  0.18 (0.13-0.27)  0.22 (0.16-0.28)  1.46 (1.15-1.83)  0.93 (0.73-1.18)  0.43 (0.33-0.55)  1.12 (0.74-1.92)  2.20 (1.70-2.80)  9 (6-16)  49 (35-63)  83 (71-88)  2 (1-3)  40662 (32643-48853)  19%  9.72 (8.35-11.27)  1.63 (1.23-2.02)  1.09 (0.73-1.54)  21.20 (9.90-71.60) | 103  83  100  103  83  86  86  92  92  100  92  92  92  92  92  92  92  85  83  85  85  91  85 (16)  103  103  103  99 | r: 0.016; p: 0.871  **r: -0.309; p: 0.005***  r: 0.018; p. 0.856  r: 0.031; p: 0.758  r: 0.177; p: 0.109  r: 0.025; p: 0.819  r: -0.052; p: 0.634  **r: -0.249; p: 0.017***  r: -0.170; p: 0.105  r: -0.044; p: 0.677  r: -0.137; p: 0.192  r: -0.169; p: 0.107  **r: -0.207; p: 0.048***  **r: -0.221; p: 0.034***  r: -0.088; p: 0.403  **r: 0.270; p: 0.009***  r: -0.142; p: 0.177  **r: 0.299; p: 0.005***  r: 0.179; p: 0.105  r: -0.141; p: 0.198  r: 0.086; p. 0.432  r: 0.100; p: 0.346  p: 0.982  r: -0.151; p: 0.128  r: 0.007; p: 0.947  r: -0.103; p: 0.300  r: -0.067; p: 0.510 | >0.999  0.252  >0.999  >0.999  >0.999  >0.999  >0.999  0.498  >0.999  >0.999  >0.999  >0.999  0.986  0.835  >0.999  0.346  >0.999  0.252  >0.999  >0.999  >0.999  >0.999  >0.999  >0.999  >0.999  >0.999 | 1.40 (0.60-3.00)  280.00 (160-434)  344.00 (234-492)  68.90 (43-114)  27.70 (20-36)  1040 (930-1225)  230 (190-300)  1.95 (1.60-2.25)  0.41 (0.34-0.57)  4.00 (3.17-5.09)  0.17 (0.14-0.28)  0.22 (0.17-0.30)  1.43 (1.15-1.70)  0.94 (0.81-1.16)  0.39 (0.29-0.54)  1.48 (0.84-2.13)  2.65 (1.90-2.90)  15 (9-24)  57 (46-69)  73 (60-88)  2 (1-4)  42027 (35133-48189)  3%  9.81 (8.75-10.77)  1.61 (1.15-2.32)  1.23 (0.79-1.65)  24.05 (12.68-98.98) | 39  37  39  39  37  37  37  38  38  38  38  38  38  38  38  38  38  37  37  37  37  38  35 (1)  39  39  39  38 | r: 0.010; p: 0.954  r: 0.095; p: 0576  r: 0.071; p: 0.669  r: 0.007; p: 0.966  r: 0.302; p: 0.069  r: -0.113; p: 0.506  r: -0.025; p: 0.882  r: -0.153; p: 0.358  r: -0.178; p: 0.285  r: -0.089; p: 0.594  r: -0.118; p: 0.481  r: 0.282; p: 0.086  r: -0.202; p: 0.223  r: -0.268; p: 0.104  r: -0.069; p: 0.681  r: 0.127; p: 0.448  r: -0.112; p: 0.504  r: 0.297; p: 0.074  r: 0.320; p. 0.054  r: -0.226; p: 0.179  r: 0.134; p: 0.430  r: 0.034; p: 0.841  p: 0.743  r: -0.040; p: 0.811  **r: -0.366; p: 0.022***  r: 0.160; p: 0.330  r: -0.263; p: 0.111 | >0.999  >0.999  >0.999  >0.999  >0.999  >0.999  >0.999  >0.999  >0.999  >0.999  >0.999  >0.999  >0.999  >0.999  >0.999  >0.999  >0.999  >0.999  >0.999  >0.999  >0.999  >0.999  >0.999  >0.999  >0.999  >0.999 |
| Markers of organ function:^1^  LDH  CK  HbA1c  GPT  GOT  GGT  Albumin  Bilirubin  Creatinine  Total protein  NTproBNP | U/l  U/l  %  U/l  U/l  U/l  g/l  mg/dl  mg/dl  g/l  ng/l | 225 (204-259)  74 (58-94)  5.20 (5.00-5.40)  19.00 (15-25)  23.00 (20-26)  15.00 (12-21)  45.20 (44-47)  0.35 (0.28-0.48)  0.72 (0.65-0.79)  0.71 (0.68-0.75)  51 (26-82) | 143  138  134  143  143  143  134  137  145  122  135 | r: 0.121; p: 0.149  **r: 0.268; p: 0.001***  r: 0.136; p: 0.118  **r: 0.197; p: 0.018***  r: 0.112; p: 0.182  **r: 0.170; p: 0.042***  r: 0.005; p: 0.957  r: -0.024; p: 0.784  r: 0.153; p: 0.066  r: -0.008; p: 0.929  r: 0.074; p: 0.394 | >0.999  0.198  >0.999  0.489  >0.999  0.870  >0.999  >0.999  >0.999  >0.999  >0.999 | 227 (201-254)  73.50 (58-92)  5.20 (4.90-5.30)  18.00 (15-25)  23.00 (19-25)  14.00 (11-20)  44.90 (44-47)  0.37 (0.28-0.52)  0.73 (0.65-0.80)  71.00 (68-75)  53.00 (26-82) | 103  100  96  103  103  103  96  99  105  84  96 | **r: 0.228; p: 0.020***  **r: 0.329; p: 0.001***  r: 0.195; p: 0.057  **r: 0.271; p: 0.006***  **r: 0.252; p: 0.010***  **r: 0.203; p: 0.040***  r: 0.075; p: 0.467  r: 0.046; p: 0.654  r: 0.115; p: 0.243  r: 0.064; p: 0.565  r: 0.019; p: 0.852 | 0.551  0.221  >0.999  0.252  0.346  0.890  >0.999  >0.999  >0.999  >0.999  >0.999 | 224 (213-289)  75.00 (59-95)  5.20 (5.08-5.40)  21.00 (16-33)  24.00 (22-27)  17.00 (12-24)  45.30 (44-46)  0.30 (0.27-0.41)  0.71 (0.63-0.77)  71.00 (69-73)  50.00 (23-76) | 39  38  38  39  39  39  38  38  39  37  39 | r: -0.149; p: 0.364  r: 0.116; p. 0.487  r: -0.031; p: 0.852  r: 0.044; p: 0.792  r: -0.223; p: 0.172  r: 0.041; p. 0.805  r: -0.175; p: 0.292  r: -0.259; p: 0.116  r: 0.269; p: 0.097  r: -0.230; p: 0.172  r: 0.133; p: 0.421 | >0.999  >0.999  >0.999  >0.999  >0.999  >0.999  >0.999  >0.999  >0.999  >0.999  >0.999 |
| Electrolytes:  Sodium  Potassium  Calcium (Albumin-corrected) ^2^  Phosphate  Zinc | mmol/l  mmol/l  mmol/l  mmol/l  µmol/l | 141.00 (139-142)  3.90 (3.70-4.00)  2.23 (2.18-2.28)  1.02 (0.90-1.13)  12.80 (11.53-14.28) | 143  143  133  136  132 | r: 0.118; p: 0.159  r: -0.067; p: 0.423  r: 0.114; p: 0.190  r: 0.067; p: 0.441  r: 0.061; p: 0.486 | >0.999  >0.999  >0.999  >0.999  >0.999 | 141.00 (139-142)  3.90 (3.70-4.10)  2.24 (2.17-2.28)  1.03 (0.93-1.13)  12.50 (11.50-14.20) | 103  103  95  99  93 | r: 0.117; p: 0.240  r: -0.126; p: 0.206  r: 0.147; p: 0.155  r: -0.054; p: 0.597  r: 0.082; p: 0.437 | >0.999  >0.999  >0.999  >0.999  >0.999 | 141.00 (139-142)  3.80 (3.70-4.00)  2.23 (2.19-2.27)  0.97 (0.87-1.11)  13.30 (11.60-14.65) | 39  38  38  37  38 | r: 0.127; p: 0.441  r: 0.080; p: 0.628  r: 0.038; p: 0.820  **r: 0.353; p: 0.032***  r: -0.078; p: 0.642 | >0.999  >0.999  >0.999  >0.999  >0.999 |
| Thyroid assessment:  fT3  fT4  TSH basal  Thyroid peroxidase (TPO)-AAB  Thyrotropin receptor (TR)-AAB  Thyroglobulin (Tg)-AAB | ng/l  ng/l  mU/l  kU/l  kU/l  U/l | 3.08 (2.86-3.39)  12.44 (11.65-13.96)  1.31 (0.94-1.98)  9.00 (8.00-13.00)  0.89 (0.89-0.89)  11.40 (10.00-21.30) | 71  72  135  72  71  71 | r: 0.146; p: 0.225  r: -0.116; p: 0.330  r: 0.020; p: 0.822  r: 0.061; p: 0.612  r: 0.013; p: 0.913  r: 0.129; p: 0.284 | >0.999  >0.999  >0.999  >0.999  >0.999  >0.999 | 3.17 (2.86-3.51)  12.29 (11.70-13.95)  1.32 (1.00-1.98)  9.00 (8.00-13.00)  0.89 (0.89-0.89)  10.30 (10.00-17.65) | 48  49  99  49  48  48 | r: 0.216; p: 0.141  r: -0.072; p: 0.621  r: 0.007; p: 0.942  r: 0.062; p: 0.671  r: 0.197; p: 0.179  r: 0.118; p: 0.424 | >0.999  >0.999  >0.999  >0.999  >0.999  >0.999 | 2.98 (2.86-3.27)  12.44 (11.43-13.25)  1.13 (0.82-1.74)  9.00 (8.00-16.75)  0.89 (0.89-0.89)  15.75 (10.00-240.93) | 22  22  36  22  22  22 | r: -0.019; p: 0.934  r: -0.295; p: 0.182  r: 0.089; p: 0.606  r: 0.031; p: 0.892  r: -0.330; p: 0.133  r: 0.009; p: 0.970 | >0.999  >0.999  >0.999  >0.999  >0.999  >0.999 |
| Autoantibodies:  alpha1-AR-AAB  alpha2-AR-AAB  beta1-AR-AAB  beta2-AR-AAB  beta3 -AR-AAB  M3-mAChR-AAB  M4-mAChR-AAB  AT1-R-AAB  ETA-R-AAB  ETB-R-AAB | U/ml  U/ml  U/ml  U/ml  U/ml  U/ml  U/ml  U/ml  U/ml  U/ml | 7.60 (5.38-10.52)  9.09 (6.93-12.73)  8.96 (5.98-12.73)  6.64 (4.76-10.11)  7.46 (5.23-11.47)  4.47 (3.40-6.13)  7.20 (5.59-9.89)  11.72 (9.47-16.91)  9.73 (7.58-12.45)  13.06 (10.44-17.79) | 141  143  143  145  142  144  144  145  145  141 | **r: -0.183; p: 0.030***  r: 0.004; p: 0.963  r: -0.150; p: 0.074  r: -0.065; p: 0.439  r: -0.045; p: 0.594  **r: -0.231; p: 0.005***  r: -0.008; p: 0.928  r: 0.042; p: 0.617  r: 0.023; p: 0.781  r: -0.061; p: 0.470 | 0.736  >0.999  >0.999  >0.999  >0.999  0.436  >0.999  >0.999  >0.999  >0.999 | 7.59 (5.43-10.80)  8.71 (6.47-12.32)  9.12 (5.97-14.14)  6.64 (4.65-11.02)  7.39 (5.23-11.50)  4.54 (3.42-6.43)  7.28 (5.47-9.82)  11.50 (9.34-16.26)  9.05 (7.47-12.10)  13.21 (10.47-18.43) | 102  103  103  105  102  105  105  105  105  101 | **r: -0.286; p: 0.004***  r: -0.060; p: 0.549  r: -0.161; p: 0.103  r: -0.127; p: 0.196  r: -0.054; p: 0.587  **r: -0.303; p: 0.002***  r: -0.086; p: 0.385  r: -0.069; p: 0.487  r: -0.023; p: 0.813  r: -0.099; p: 0.323 | 0.252  >0.999  >0.999  >0.999  >0.999  0.226  >0.999  >0.999  >0.999  >0.999 | 7.61 (4.96-9.79)  9.27 (7.36-13.05)  8.92 (6.05-11.62)  6.45 (5.00-9.48)  7.55 (5.14-11.07)  4.10 (3.25-6.09)  6.93 (5.76-10.64)  11.74 (9.51-17.80)  10.11 (8.11-12.99)  12.21 (10.39-15.80) | 38  39  39  39  39  38  38  39  39  39 | r: 0.023; p. 0.889  r: 0.141; p: 0.391  r: -0.111; p: 0.503  r: 0.118; p: 0.473  r: -0.005; p: 0.977  r: -0.045; p: 0.787  r: 0.139; p: 0.404  r: 0.301; p: 0.063  r: 0.117; p: 0.477  r: 0.094; p: 0.570 | >0.999  >0.999  >0.999  >0.999  >0.999  >0.999  >0.999  >0.999  >0.999  >0.999 |

^1^ LDH (females): 135-250U/l, CK (females): <167U/l, HbA1c: <6%, GPT (females): <31U/l, GOT (females): <35U/l, GGT (females): 5-36U/l, Albumin: 35-52g/l, Bilirubin: <1.2mg/dl, Creatinine (females): 0.5-0.9mg/dl, Total protein: 64-83g/l, NTproBNP: <97ng/l (up to 44a; higher in older age)

^2^ Albumin-correction for Calcium was performed as: Calcium_corrected_ (mmol/l) = Calcium_total_ (mmo/l) – (0.025 x Albumin (g/l)) + 1

**Table S2(C)**: Correlations between sCD26 and Schellong examination outcome in female ME/CFS patients (onset stratified)

| parameter | | Female patients (n=145) | | | | Female patients with infection triggered onset (n=105) | | | | Female patients with non-infection-triggered onset (n=39) | | | |
| --- | --- | --- | --- | --- | --- | --- | --- | --- | --- | --- | --- | --- | --- |
|  |  | median (IQR)/% | n (criteria pos) | Correlation/Mann-U | BY-corrected p | median (IQR)/% | n (criteria pos) | Correlation/Mann-U | BY-corrected p | median (IQR)/% | n (criteria pos) | Correlation/Mann-U | BY-corrected p |
| POTS |  | 14% | 131 (22) | p: **0.049*** (sCD26 lower in POTS) |  | 18% | 94 (17) | p: 0.406 |  | 14% | 36 (5) | **p: 0.021*** (sCD26 lower in POTS) |  |
| Schellong examination:  heart rate  blood pressure (sys)  blood pressure (dia) | /min  mmHg  mmHg | 81 (75-93)  128 (116-142)  87 (81-97) | 132  131  131 | r: -0.078; p: 0.373  r: 0.108; p: 0.218  r: 0.062; p. 0.479 | >0.999  >0.999  >0.999 | 81 (75-91)  124 (116-139)  86 (81-96) | 95  94  94 | r: 0.056; p: 0.589  r: 0.141; p: 0.174  r: 0.089; p: 0.393 | >0.999  >0.999  >0.999 | 83 (71-96)  138.00 (122-144)  92 (82-98) | 36  36  36 | **r: -0.445; p: 0.007***  r: 0.050; p: 0.770  r: -0.054; p: 0.753 | 0.131  >0.999  >0.999 |
| heart rate standing 0min  heart rate after 2min  heart rate after 5min  heart rate after 10min  blood pressure (sys) standing 0min  blood pressure (dia) standing 0min  blood pressure (sys) after 2min  blood pressure (dia) after 2min  blood pressure (sys) after 5min  blood pressure (dia) after 5min  blood pressure (sys) after 10min  blood pressure (dia) after 10min  changes heartrate seated🡪standing  changes RR (sys) seated🡪standing  changes RR (dia) seated🡪standing | /min  /min  /min  /min  mmHg  mmHg  mmHg  mmHg  mmHg  mmHg  mmHg  mmHg  /min  mmHg  mmHg | 91 (81-98)  91 (83-101)  91 (81-101)  96 (87-104)  126 (115-143)  89 (83-101)  125 (117-141)  91 (84-102)  125 (117-137)  92 (86-99)  123 (112-135)  91 (84-103)  7 (2-14)  -1 (-9-7)  2 (-2-8) | 130  129  128  82  129  129  127  127  128  128  81  82  130  129  129 | **r: -0.200; p: 0.023***  **r: -0.186; p: 0.035***  **r. -0.197; p: 0.026***  r: -0.161; p: 0.148  r: 0.140; p: 0.114  r: -0.004; p: 0.967  r: 0.135; p: 0.131  r: -0.011; p: 0.900  r: 0.150; p: 0.090  r: 0.108; p. 0.225  r: 0.065; p. 0.563  r: -0.072; p: 0.522  **r: -0.231; p. 0.008***  r: 0.065; p: 0.464  r. -0.098; p: 0.270 | 0.544  0.551  0.544  >0.999  >0.999  >0.999  >0.999  >0.999  >0.999  >0.999  >0.999  >0.999  0.521  >0.999  >0.999 | 90 (83-98)  91 (83-101)  91 (85-101)  96 (87-103)  125 (114-139)  88 (82-100)  123 (116-138)  90 (84-100)  124 (116-135)  90 (86-98)  123 (112-133)  90 (83-101)  8 (2-14)  -1 (-8-7)  2 (-2-7) | 94  92  91  62  93  93  90  90  91  91  62  62  94  93  93 | r: -0.065; p: 0.535  r: -0.063; p: 0.549  r: -0.023; p: 0.826  r: -0.048; p: 0.713  r: 0.150; p: 0.150  r: 0.027; p: 0.795  r: 0.181; p: 0.088  r: -0.002; p: 0.984  r: 0.199; p: 0.059  r: 0.163; p: 0.122  r: 0.054; p: 0.675  r: -0.009; p: 0.944  **r: -0.211; p: 0.041***  r: 0.002; p: 0.988  r: -0.100; p: 0.339 | >0.999  >0.999  >0.999  >0.999  >0.999  >0.999  >0.999  >0.999  >0.999  >0.999  >0.999  >0.999  >0.999  >0.999  >0.999 | 91 (75-101)  89 (77-101)  91 (76-106)  98 (87-112)  131 (122-150)  94 (86-104)  131 (118-154)  96 (85-106)  134 (120-150)  94 (84-105)  123 (114-152)  94 (85-103)  7 (2-12)  -3 (-11-7)  4 (0-9) | 35  36  36  19  35  35  36  36  36  36  19  20  35  35  35 | **r: -0.566; p: <0.001***  **r: -0.433; p: 0.008***  **r: -0.558; p: <0.001***  **r: -0.501; p: 0.025***  r: 0.134; p: 0.441  r: -0.061; p: 0.729  r: 0.040; p: 0.819  r: -0.001; p: 0.995  r: 0.054; p: 0.756  r: -0.032; p: 0.851  r: 0.112; p: 0.647  r: -0.212; p: 0.369  r: -0.203; p: 0.243  r: 0.228; p: 0.187  r: -0.125; p: 0.474 | 0.013  0.131  0.013  0.309  >0.999  >0.999  >0.999  >0.999  >0.999  >0.999  >0.999  >0.999  >0.999  >0.999  >0.999 |

**Table S2(D)**: Correlations between sCD26 and clinical questionnaire outcome in female patients (onset stratified)

| parameter | | Female patients (n=145) | | | | Female patients with infection triggered onset (n=105) | | | | Female patients with non-infection-triggered onset (n=39) | | | |
| --- | --- | --- | --- | --- | --- | --- | --- | --- | --- | --- | --- | --- | --- |
|  |  | median (IQR)/% | n (criteria pos) | Correlation/Mann-U | BY-corrected p | median (IQR)/% | n (criteria pos) | Correlation/Mann-U | BY-corrected p | median (IQR)/% | n (criteria pos) | Correlation/Mann-U | BY-corrected p |
| Clinical questionnaire outcome:  fatigue  fatigue score  cognitive score  muscle pain  immune score  disease severity |  | 8.00 (7.00-9.00)  8.25 (7.50-9.25)  7.00 (5.67-8.00)  7.00 (5.00-8.00)  5.67 (4.17-7.00)  8.00 (7.00-8.50) | 128  129  127  129  129  108 | r: 0.042; p: 0.634  r: 0.093; p: 0.294  r: 0.027; p: 0.764  r: 0.014; p: 0.878  r: 0.141; p: 0.110  r: 0.114; p: 0.240 | >0.999  >0.999  >0.999  >0.999  >0.999  >0.999 | 8.00 (7.00-9.00)  8.00 (7.50-9.14)  7.00 (5.67-7.83)  7.00 (5.00-8.00)  5.67 (4.33-7.00)  8.00 (7.00-8.38) | 93  94  92  94  94  76 | r: 0.047; p: 0.656  r: 0.119; p: 0.255  r: -0.019; p: 0.860  r: 0.026; p: 0.805  **r: 0.241; p: 0.019***  r: 0.136; p: 0.242 | >0.999  >0.999  >0.999  >0.999  0.560  >0.999 | 9.00 (8.00-10.00)  8.57 (7.75-9.41)  7.00 (5.59-8.00)  8.00 (6.00-8.00)  5.33 (3.92-6.53)  8.00 (7.00-9.00) | 34  34  34  34  34  31 | r: 0.065; p: 0.714  r: 0.054; p: 0.760  r: 0.171; p: 0.332  r: 0.007; p: 0.968  r: -0.051; p: 0.776  r: 0.121; p: 0.518 | >0.999  >0.999  >0.999  >0.999  >0.999  >0.999 |
| Bell score |  | 30.00 (30.00-40.00) | 145 | r: -0.101; p: 0.225 | >0.999 | 30.00 (30.00-40.00) | 105 | r: -0.151; p: 0.123 | >0.999 | 30.00 (30.00-40.00) | 39 | r: 0.097; p: 0.558 | >0.999 |
| COMPASS 31 score total |  | 45.73 (37.49-54.09) | 135 | r: -0.005; p: 0.953 | >0.999 | 45.49 (34.55-52.86) | 98 | r: 0.009; p: 0.928 | >0.999 | 49.72 (39.51-56.73) | 36 | r: <0.001; p: 0.999 | >0.999 |
| Chalder score |  | 27.00 (25.00-30.00) | 136 | r: 0.008; p: 0.926 | >0.999 | 27.00 (25.00-29.00) | 99 | r: 0.007; p: 0.946 | >0.999 | 28.00 (24.00-31.00) | 36 | r: 0.016: p: 0.924 | >0.999 |
| SF-36 score |  | 35.00 (20.00-52.50) | 93 | r: -0.069; p: 0.510 | >0.999 | 30.00 (15.00-55.00) | 62 | r: -0.217; p: 0.091 | >0.999 | 35.00 (20.00-46.25) | 30 | r: 0.150; p: 0.430 | >0.999 |

**Table S3(A)**: Correlations between sCD26 and group characteristics in male ME/CFS patients (onset stratified)

| parameter | | Male patients (n=60) | | | Male patients with infection triggered onset (n=41) | | | Male patients with non-infection-triggered onset (n=18) | | |
| --- | --- | --- | --- | --- | --- | --- | --- | --- | --- | --- |
|  |  | median (IQR)/% | n (criteria pos) | Correlation/Mann-U | median (IQR)/% | n (criteria pos) | Correlation/Mann-U | median (IQR)/% | n (criteria pos) | Correlation/Mann-U |
| age | a | 41 (30-50) | 60 | r: -0.115; p: 0.382 | 40 (30.5-46.5) | 41 | r: -0.023; p: 0.889 | 49 (29-54) | 18 | r: -0.026; p: 0.919 |
| Infection-triggered disease onset |  | 69% | 59 (41) | **p: 0.035*** (higher in inf. onset) | - | - | - | - | - | - |
| onset trigger Ebstein-Barr virus |  | 12% | 60 (7) | p: 0.143 | 17% | 41 (7) | p: 0.349 | - | - | - |
| disease duration | a | 4 (2-8) | 60 | r: -0.179; p: 0.172 | 4 (2-8) | 41 | r: -0.297; p: 0.059 | 4,5 (1-8) | 18 | r: 0.068; p: 0.788 |

**Table S3(B)**: Correlations between sCD26 and laboratory assessments in male ME/CFS patients (onset stratified)

| parameter | | Male patients (n=60) | | | | Male patients with infection triggered onset (n=41) | | | | Male patients with non-infection-triggered onset (n=18) | | | |
| --- | --- | --- | --- | --- | --- | --- | --- | --- | --- | --- | --- | --- | --- |
|  |  | median (IQR)/% | n (criteria pos) | Correlation/Mann-U | BY-corrected p | median (IQR)/% | n (criteria pos) | Correlation/Mann-U | BY-corrected p | median (IQR)/% | n (criteria pos) | Correlation/Mann-U | BY-corrected p |
| Immunological assessment:  CrP  IL-1b  soluble IL-2 receptor  Ferritin  Angiotensin converting enzyme  C3 complement  C4 complement  lymphocytes  monocytes  granulocytes  natural killer (NK) cells  CD19+ B cells  CD3+ T cells  CD4+ T cells  CD8+ T cells  CD4+CD8+ T cells  CD4/CD8-ratio  HLA-DR+/CD8+ T cells  CD11a+/CD8- T cells  CD28+/CD8+ T cells  CD57+/CD8- T cells  monocytal HLA-DR expression  ANA >1:160  IgG  IgA  IgM  IgE | mg/dl  pg/ml  IU/ml  µg/ml  U/l  mg/dl  mg/dl  /nl  /nl  /nl  /nl  /nl  /nl  /nl  /nl  %  %  %  %  %  ab/cell  g/l  g/l  g/l  kU/l | 2.70 (0.60-3.00)  197.00 (142-323)  340.50 (312-447)  120.55 (88-188)  27.00 (17-38)  1030 (880-1210)  230 (200-300)  1.78 (1.34-2.23)  0.47 (0.38-0.61)  4.17 (3.312-5.45)  0.20 (0.13-0.27)  0.22 (0.16-0.31)  1.29 (0.96-1.60)  0.78 (0.62-1.09)  0.38 (0.28-0.51)  1.03 (0.70-1.76)  2.10 (1.53-2.60)  14 (6-21)  50 (39-60)  81 (69-89)  2 (1-4)  37421 (31882-47587)  11%  9.55 (8.00-11.02)  1.92 (1.41-2.51)  0.90 (0.65-1.16)  45.00 (13.00-102.00) | 59  50  56  60  47  47  47  52  52  52  52  52  52  52  52  52  52  50  50  50  50  52  53 (6)  60  60  60  58 | r: 0.042; p: 0.754  r: -0.160; p: 0.267  r: -0.087; p: 0.522  r: 0.029; p: 0.828  r: -0.180; p: 0.225  r: 0.139; p: 0.352  r: 0.055; p: 0.715  r: 0.021; p: 0.882  r: 0.076; p: 0.593  r: -0.148; p: 0.295  r: -0.100; p: 0.482  r: 0.019; p: 0.892  r: -0.008; p: 0.957  r: -0.051; p: 0.722  r: 0.042; p: 0.766  r: 0.040; p: 0.779  r: -0.104; p: 0.464  **r: 0.359; p: 0.010***  r: -0.013; p: 0.930  r: -0.079; p: 0.586  r: -0.022; p: 0.881  r: -0.184; p: 0.191  p: 0.967  r: 0.201; p: 0.124  r: 0.183; p: 0.163  r: -0.077; p: 0.557  r: 0.106; p: 0.430 | >0.999  >0.999  >0.999  >0.999  >0.999  >0.999  >0.999  >0.999  >0.999  >0.999  >0.999  >0.999  >0.999  >0.999  >0.999  >0.999  >0.999  0.917  >0.999  >0.999  >0.999  >0.999  >0.999  >0.999  >0.999  >0.999 | 1.90 (0.43-3.00)  196.00 (141-317)  335.00 (311-403)  120.40 (93-173)  28.15 (16-37)  1040 (905-1208)  220 (200-308)  1.73 (1.33-2.05)  0.45 (0.33-0.62)  4.03 (3.06-5.38)  0.19 (0.13-0.27)  0.20 (0.16-0.29)  1.27 (0.93-1.57)  0.76 (0.62-1.07)  0.33 (0.28-0.47)  0.98 (0.67-1.45)  2.10 (1.60-2.85)  14 (7-20)  49 (40-60)  79 (69-88)  2 (1-3)  38331 (32102-47199)  8%  10.00 (8.45-11.12)  2.02 (1.51-2.49)  0.94 (0.66-1.14)  41.35 (13.43-79.38) | 40  35  38  41  32  32  32  37  37  41  37  37  37  37  37  37  37  35  35  35  35  37  36 (4)  41  41  41  40 | r: 0.073; p: 0.655  r: -0.223; p: 0.199  r: 0.139; p: 0.406  r: 0.186; p: 0.245  **r: -0.369; p: 0.038***  r: 0.017; p: 0.925  r: 0.043; p: 0.815  r: -0.011; p: 0.947  r: 0.230; p: 0.171  r: -0.039; p: 0.817  r: -0.034; p: 0.843  r: 0.024; p: 0.890  r: -0.063; p: 0.713  r: -0.076; p: 0.653  r: -0.047; p: 0.781  r: -0.070; p: 0.681  r: -0.059; p: 0.727  **r: 0.377; p: 0.026***  r: 0.006; p: 0.973  r: -0.147; p: 0.400  r: -0.032; p: 0.853  r: -0.240; p. 0.153  p: 0.865  r: 0.130; p. 0.416  r: 0.268; p. 0.090  r: -0.131; p: 0.415  r: 0.249; p: 0.121 | >0.999  >0.999  >0.999  >0.999  >0.999  >0.999  >0.999  >0.999  >0.999  >0.999  >0.999  >0.999  >0.999  >0.999  >0.999  >0.999  >0.999  >0.999  >0.999  >0.999  >0.999  >0.999  >0.999  >0.999  >0.999  >0.999 | 3.00 (0.95-3.00)  211.50 (131-359)  383.00 (305-513)  126.75 (86-205)  25.30 (20-40)  1010 (805-1180)  240 (215-293)  2.20 (1.41-2.37)  0.51 (0.42-0.66)  4.83 (3.80-5.78)  0.23 (0.17-0.27)  0.33 (0.17-0.37)  1.51 (1.08-1.86)  1.03 (0.65-1.24)  0.42 (0.29-0.55)  1.15 (0.77-2.66)  2.15 (1.40-2.55)  10 (4-27)  55 (29-65)  87 (61-92)  2 (1-8)  36761 (32744-48771)  13%  8.98 (7.53-9.74)  1.50 (1.23-2.76)  0.81 (0.62-1.24)  71.40 (15.00-187.00) | 18  14  17  18  14  14  14  14  14  14  14  14  14  14  14  14  14  14  14  14  14  14  16 (2)  18  18  18  17 | r: 0.382; p: 0.118  r: 0.218; p: 0.454  r: -0.468; p: 0.058  r: -0.366; p: 0.135  r: 0.209; p: 0.474  r: 0.327; p: 0.253  r: 0.301; p: 0.296  r: 0.167; p: 0.567  r: -0.156; p: 0.594  r: -0.459; p: 0.098  r: 0.022; p: 0.940  r: 0.366; p: 0.198  r: 0.112; p: 0.703  r: -0.084; p: 0.776  r: 0.187; p: 0.523  r: 0.477; p: 0.085  r: -0.329; p: 0.250  r: 0.108; p: 0.713  r: -0.090; p: 0.759  r: 0.163; p: 0.578  r: 0.098; p: 0.738  r: 0.095; p: 0.748  p: 0.600  r: 0.255; p: 0.307  r: -0.055; p: 0.829  r: 0.034; p: 0.893  r: 0.132; p: 0.612 | >0.999  >0.999  >0.999  >0.999  >0.999  >0.999  >0.999  >0.999  >0.999  >0.999  >0.999  >0.999  >0.999  >0.999  >0.999  >0.999  >0.999  >0.999  >0.999  >0.999  >0.999  >0.999  >0.999  >0.999  >0.999  >0.999  >0.999 |
| Markers of organ function:^1^  LDH  CK  HbA1c  GPT  GOT  GGT  Albumin  Bilirubin  Creatinine  Total protein  NTproBNP | U/l  U/l  %  U/l  U/l  U/l  g/l  mg/dl  mg/dl  g/l  ng/l | 234 (218-268)  118 (93-162)  5.10 (4.80-5.40)  28.00 (22-48)  27.00 (23-33)  22.00 (15-43)  46.90 (45-49)  0.53 (0.33-67)  0.92 (0.82-1.00)  72.00 (69-75)  27.00 (10-40) | 58  57  55  59  59  59  55  58  60  47  55 | **r: 0.317; p: 0.015***  r: -0.047; p: 0.731  r: 0.142; p: 0.301  **r: 0.354; p: 0.006***  **r: 0.367; p: 0.004***  **r: 0.259; p: 0.047***  r: 0.168; p: 0.220  r: 0.050; p: 0.708  r: 0.203; p: 0.120  r: 0.073; p: 0.626  r: -0.143; p: 0.296 | >0.999  >0.999  >0.999  0.777  0.777  >0.999  >0.999  >0.999  >0.999  >0.999  >0.999 | 236 (220-273)  121.00 (94-163)  5.00 (4.80-5.35)  28.50 (22-48)  27.00 (23-34)  22.00 (15-43)  47.00 (45-49)  0.57 (0.37-0.72)  0.92 (0.84-1.00)  72.50 (70-76)  24.00 (10-35) | 39  39  37  40  40  40  37  40  41  32  37 | **r: 0.369; p: 0.021***  r: -0.019; p: 0.911  **r: 0.360; p: 0.029***  **r: 0.357; p: 0.024***  **r: 0.385; p: 0.014***  **r: 0.355; p: 0.024***  r: 0.242; p: 0.149  r: 0.011; p. 0.948  r: 0.199; p: 0.213  r: -0.050; p: 0.786  r: -0.120; p: 0.478 | >0.999  >0.999  >0.999  >0.999  >0.999  >0.999  >0.999  >0.999  >0.999  >0.999  >0.999 | 228 (201-245)  118.00 (91-162)  5.30 (5.00-5.70)  24.00 (21-38)  26.00 (20-32)  20.00 (16-45)  46.70 (45-49)  0.36 (0.32-0.53)  0.88 (0.79-1.00)  70.50 (67-74)  34.00 (12-88) | 18  17  17  18  18  18  17  17  18  14  17 | r: 0.063; p: 0.804  r: 0.009; p: 0.974  r: 0.065; p: 0.804  r: 0.327; p: 0.186  r: 0.265; p: 0.287  r: 0.044; p: 0.861  r: 0.006; p: 0.981  r: -0.231; p: 0.373  r: 0.095; p. 0.707  r: 0.278; p: 0.336  r: -0.287; p: 0.264 | >0.999  >0.999  >0.999  >0.999  >0.999  >0.999  >0.999  >0.999  >0.999  >0.999  >0.999 |
| Electrolytes:  Sodium  Potassium  Calcium (Albumin-corr.) ^2^  Phosphate  Zinc | mmol/l  mmol/l  mmol/l  mmol/l  µmol/l | 141.00 (140-142)  4.00 (3.80-4.30)  2.21 (2.17-2.28)  0.94 (0.79-1.06)  14.30 (12.50-15.55) | 58  58  55  57  53 | r: -0.239; p: 0.071  r: -0.232; p: 0.079  r: 0.260; p: 0.056  r: -0.002; p: 0.986  r: -0.001; p: 0.994 | >0.999  >0.999  >0.999  >0.999  >0.999 | 141.00 (140-142)  3.90 (3.80-4.10)  2.21 (2.17-2.28)  0.87 (0.76-1.06)  14.35 (12.85-16.43) | 40  40  37  39  38 | r: -0.285; p: 0.074  r: -0.071; p: 0.663  r: 0.161; p: 0.341  r: 0.056; p: 0.734  r: -0.122; p: 0.464 | >0.999  >0.999  >0.999  >0.999  >0.999 | 141.00 (140-143)  4.20 (3.95-4.40)  2.19 (2.16-2.25)  1.01 (0.92-1.09)  13.50 (11.68-14.93) | 17  17  17  17  14 | r: -0.202; p: 0.437  **r: -0.582; p: 0.014***  r: 0.456; p: 0.066  r: 0.119; p: 0.649  r: 0.200; p: 0.493 | >0.999  >0.999  >0.999  >0.999  >0.999 |
| Thyroid assessment:  fT3  fT4  TSH basal  Thyroid peroxidase (TPO)-AAB  Thyrotropin receptor (TR)-AAB  Thyroglobulin (Tg)-AAB | ng/l  ng/l  mU/l  kU/l  kU/l  U/l | 3.50 (3.13-3.79)  12.92 (11.67-14.84)  1.60 (1.10-2.21)  9.00 (9.00-11.00)  0.89 (0.89-0.89)  10.00 (10.00-15.50) | 32  32  57  35  33  31 | r: 0.118; p: 0.521  r: -0.232; p: 0.202  r: 0.104; p: 0.441  r: -0.054; p: 0.759  ---  r: -0.053; p: 0.776 | >0.999  >0.999  >0.999  >0.999  ---  >0.999 | 3.57 (3.25-3.90)  13.08 (11.62-14.95)  1.76 (1.05-2.39)  9.00 (9.00-11.00)  0.89 (0.89-0.89)  10.00 (10.00-10.90) | 20  20  40  22  20  19 | r: 0.025; p. 0.917  r: -0.220; p. 0.352  r: 0.052; p. 0.751  r: 0.202; p: 0.368  ---  r: -0.049; p: 0.842 | >0.999  >0.999  >0.999  >0.999  ---  >0.999 | 3.23 (2.96-3.63)  12.05 (11.77-14.48)  1.30 (1.13-1.94)  10.00 (7.25-12.50)  0.89 (0.89-0.89)  12.40 (10.00-16.10) | 11  11  16  13  12  11 | r: 0.255; p: 0.450  r: -0.209; p: 0.537  r: 0.191; p: 0.478  **r: -0.657; p: 0.020***  **---**  r: -0.010; p: 0.978 | >0.999  >0.999  >0.999  >0.999  ---  >0.999 |
| Autoantibodies:  alpha1-AR-AAB  alpha2-AR-AAB  beta1-AR-AAB  beta2-AR-AAB  beta3 -AR-AAB  M3-mAChR-AAB  M4-mAChR-AAB  AT1-R-AAB  ETA-R-AAB  ETB-R-AAB | U/ml  U/ml  U/ml  U/ml  U/ml  U/ml  U/ml  U/ml  U/ml  U/ml | 7.82 (4.40-10.30)  9.28 (6.84-12.36)  8.34 (6.12-12.23)  5.77 (3.88-8.26)  6.70 (4.59-11.49)  4.23 (3.14-5.48)  6.77 (5.38-9.51)  12.13 (9.41-17.40)  9.73 (7.94-12.35)  13.77 (10.20-18.49) | 59  59  59  59  58  59  59  60  60  58 | r: -0.134; p: 0.311  r: 0.018; p: 0.893  r: -0.143; p: 0.280  r: -0.143; p: 0.278  r: -0.013; p: 0.921  r: -0.103; p: 0.438  r: -0.043; p: 0.745  r: -0.041; p: 0.756  r: -0.015; p: 0.911  r: 0.019; p: 0.885 | >0.999  >0.999  >0.999  >0.999  >0.999  >0.999  >0.999  >0.999  >0.999  >0.999 | 7.72 (3.70-10.18)  9.88 (6.89-12.28)  8.59 (6.14-12.70)  5.79 (3.81-8.63)  7.32 (5.00-11.81)  4.29 (3.14-5.49)  6.71 (5.49-9.94)  12.87 (9.00-18.26)  9.82 (7.94-12.79)  14.25 (10.69-19.01) | 40  40  40  40  39  40  40  41  41  40 | r: -0.210; p. 0.193  r: -0.052; p. 0.749  **r: -0.331; p: 0.037***  r: -0.272; p: 0.089  r: -0.210; p: 0.200  r: -0.233; p: 0.148  r: -0.157; p: 0.335  r: -0.138; p: 0.390  r: -0.117; p: 0.465  r: -0.039; p. 0.810 | >0.999  >0.999  >0.999  >0.999  >0.999  >0.999  >0.999  >0.999  >0.999  >0.999 | 7.98 (4.36-10.88)  8.70 (6.69-12.42)  8.04 (5.93-10.61)  5.40 (4.23-8.46)  5.46 (4.09-9.84)  4.08 (2.68-5.48)  6.57 (4.90-8.56)  11.17 (9.36-14.37)  9.25 (7.63-11.07)  13.37 (9.24-15.84) | 18  18  18  18  18  18  18  18  18  17 | r: 0.185; p. 0.463  r: 0.139; p: 0.581  r: 0.273; p: 0.272  r: 0.220; p: 0.381  r: 0.201; p. 0.423  r: 0.230; p. 0.358  r: 0.236; p: 0.345  r: 0.203; p. 0.418  r: 0.205; p: 0.414  r: 0.199; p: 0.445 | >0.999  >0.999  >0.999  >0.999  >0.999  >0.999  >0.999  >0.999  >0.999  >0.999 |

^1^ LDH (males): 135-250U/l, CK (males): <190U/l, HbA1c: <6%, GPT (males): <41U/l, GOT (males): <50U/l, GGT (males): 8-61U/l, Albumin: 35-52g/l, Bilirubin: <1.2mg/dl, Creatinine (males): 0.7-1.2mg/dl, Total protein: 64-83g/l, NTproBNP: <97ng/l (up to 44a; higher in older age)

^2^ Albumin-correction for Calcium was performed as: Calcium_corrected_ (mmol/l) = Calcium_total_ (mmo/l) – (0.025 x Albumin (g/l)) + 1

**Table S3(C)**: Correlations between sCD26 and Schellong examination outcome in male ME/CFS patients (onset stratified)

| parameter | | Male patients (n=60) | | | | Male patients with infection triggered onset (n=41) | | | | Male patients with non-infection-triggered onset (n=18) | | | |
| --- | --- | --- | --- | --- | --- | --- | --- | --- | --- | --- | --- | --- | --- |
|  |  | median (IQR)/% | n (criteria pos) | Correlation/Mann-U | BY-corrected p | median (IQR)/% | n (criteria pos) | Correlation/Mann-U | BY-corrected p | median (IQR)/% | n (criteria pos) | Correlation/Mann-U | BY-corrected p |
| POTS |  | 15% | 48 (7) | p: 0.091 | 32 (6) | 19% |  | p: 0.055 |  | 6% | 15 (1) | p: 0.533 |  |
| Schellong examination:  heart rate  blood pressure (sys)  blood pressure (dia) | /min  mmHg  mmHg | 73 (68-85)  134 (126-148)  91 (83-98) | 50  49  49 | **r: 0.314; p: 0.027***  r: 0.233; p: 0.107  r: 0.280; p: 0.051 | 0.209  0.483  0.292 | 77 (71-93)  134 (127-149)  92 (83-98) | 34  33  33 | r: 0.290; p: 0.096  r: 0.266; p: 0.134  **r: 0.348; p: 0.047*** | 0.501  0.650  0.330 | 71 (62-76)  133.00 (124-149)  89 (85-100) | 15  15  15 | r: 0.185; p: 0.510  r: -0.004; p: 0.990  r: 0.174; p: 0.536 | >0.999  >0.999  >0.999 |
| heart rate standing 0min  heart rate after 2min  heart rate after 5min  heart rate after 10min  blood pressure (sys) standing 0min  blood pressure (dia) standing 0min  blood pressure (sys) after 2min  blood pressure (dia) after 2min  blood pressure (sys) after 5min  blood pressure (dia) after 5min  blood pressure (sys) after 10min  blood pressure (dia) after 10min  changes heartrate seated🡪standing  changes RR (sys) seated🡪standing  changes RR (dia) seated🡪standing | /min  /min  /min  /min  mmHg  mmHg  mmHg  mmHg  mmHg  mmHg  mmHg  mmHg  /min  mmHg  mmHg | 85 (78-96)  87 (80-98)  89 (78-98)  89 (79-98)  131 (121-148)  91 (85-103)  132 (121-153)  96 (87-105)  130 (124-145)  96 (87-105)  130 (121-147)  96 (81-106)  10 (5-14)  -2 (-9-4)  1 (-6-8) | 50  50  49  33  49  49  49  49  49  49  33  33  50  49  49 | r: 0.103; p. 0.475  r: 0.223; p: 0.120  r: 0.251; p: 0.082  r: 0.234; p: 0.190  **r: 0.288; p. 0.045***  **r: 0.370; p: 0.009***  **r: 0.339; p: 0.017***  **r: 0.445; p: 0.001***  **r: 0.355; p: 0.012***  **r: 0.382; p. 0.007***  **r: 0.430; p. 0.012***  **r: 0.612; p: <0.001***  **r: -0.281; p: 0.048***  r: 0.132; p. 0.365  r: 0.254; p: 0.079 | >0.999  0.504  0.397  0.746  0.292  0.131  0.153  0.042  0.131  0.131  0.131  0.010  0.292  >0.999  0.397 | 88 (81-99)  88 (81-103)  89 (82-103)  91 (83-101)  131 (117-149)  92 (84-104)  130 (121-154)  96 (87-103)  133 (123-145)  95 (87-105)  131 (119-148)  98 (84-108)  10 (5-14)  -3 (-13-4)  1 (-6-9) | 34  34  33  23  33  33  33  33  33  33  23  23  34  33  33 | r: 0.131; p: 0.462  r: 0.308; p: 0.076  **r: 0.368; p: 0.035***  r: 0.272; p: 0.210  r: 0.306; p: 0.083  **r: 0.352; p: 0.045***  **r: 0.354; p. 0.043***  **r: 0.452; p: 0.008***  **r: 0.406; p: 0.019***  **r: 0.424; p: 0.014***  **r: 0.463; p: 0.026***  **r: 0.570; p: 0.004***  r: -0.244; p: 0.165  r: 0.030; p: 0.868  r: 0.187; p. 0.296 | >0.999  0.476  0.330  0.881  0.476  0.330  0.330  0.261  0.297  0.292  0.330  0.261  0.741  >0.999  >0.999 | 83 (70-93)  83 (72-96)  85 (71-96)  81 (78-94)  128 (124-147)  88 (86-99)  132 (125-153)  91 (86-112)  130 (124-143)  96 (86-107)  125 (120-143)  81 (72-109)  13 (7-16)  0 (-7-4)  1 (-3-3) | 15  15  15  9  15  15  15  15  15  15  9  9  15  15  15 | r: -0.034; p: 0.904  r: -0.077; p: 0.785  r: -0.055; p: 0.845  r: 0.191; p: 0.622  r: 0.267; p: 0.337  r: 0.262; p: 0.346  r: 0.252; p: 0.364  r: 0.259; p: 0.351  r: 0.305; p. 0.269  r: 0.282; p: 0.308  r: -0.068; p: 0.862  r: 0.528; p: 0.144  r: -0.182; p: 0.515  **r: 0.529; p: 0.043***  r: 0.380; p: 0.162 | >0.999  >0.999  >0.999  >0.999  >0.999  >0.999  >0.999  >0.999  >0.999  >0.999  >0.999  >0.999  >0.999  >0.999  >0.999 |

**Table S3(D)**: Correlations between sCD26 and clinical questionnaire outcome in male patients (onset stratified)

| parameter | | Male patients (n=60) | | | | Male patients with infection triggered onset (n=41) | | | | Male patients with non-infection-triggered onset (n=18) | | | |
| --- | --- | --- | --- | --- | --- | --- | --- | --- | --- | --- | --- | --- | --- |
|  |  | median (IQR)/% | n (criteria pos) | Correlation/Mann-U | BY-corrected p | median (IQR)/% | n (criteria pos) | Correlation/Mann-U | BY-corrected p | median (IQR)/% | n (criteria pos) | Correlation/Mann-U | BY-corrected p |
| Clinical questionnaire outcome:  fatigue  fatigue score  cognitive score  muscle pain  immune score  disease severity |  | 8.00 (7.00-9.00)  8.44 (7.63-9.00)  7.67 (6.33-8.33)  7.00 (5.00-8.00)  4.67 (3.58-6.08)  8.00 (8.00-8.50) | 58  58  58  56  58  51 | r: -0.123; p: 0.358  r: -0.078; p: 0.563  r: -0.044; p: 0.743  r: -0.099; p: 0.466  r: 0.101; p: 0.453  **r: -0.335; p: 0.016*** | >0.999  >0.999  >0.999  >0.999  >0.999  0.480 | 8.00 (8.00-9.00)  8.50 (7.75-9.00)  8.00 (6.67-8.33)  7.00 (5.00-8.00)  4.66 (3.33-6.66)  8.00 (8.00-8.00) | 39  39  39  39  39  36 | r: -0.089; p: 0.592  r: -0.085; p: 0.608  r: -0.229; p: 0.160  r: -0.099; p: 0.550  r: 0.191; p: 0.244  **r: -0.360; p: 0.031*** | >0.999  >0.999  >0.999  >0.999  >0.999  0.908 | 8.00 (5.75-9.25)  8.13 (6.79-9.03)  7.00 (6.00-8.00)  8.00 (6.00-8.38)  5.34 (3.67-6.00)  8.25 (7.75-8.63) | 18  18  18  16  18  14 | r: -0.159; p: 0.528  r: -0.231; p: 0.357  r: 0.141; p: 0.577  r: 0.129; p: 0.634  r: 0.056; p: 0.825  r: -0.122; p: 0.679 | >0.999  >0.999  >0.999  >0.999  >0.999  >0.999 |
| Bell score |  | 30.00 (25.00-40.00) | 57 | r: 0.044; p: 0.743 | >0.999 | 30.00 (20.00-40.00) | 38 | r: -0.023; p: 0.889 | >0.999 | 35.00 (30.00-41.25) | 18 | r: 0.341; p: 0.166 | >0.999 |
| COMPASS 31 score total |  | 40.62 (29.76-48.45) | 57 | r: 0.101; p: 0.453 | >0.999 | 41.33 (30.67-51.38) | 38 | r: 0.177; p: 0.287 | >0.999 | 39.45 (23.02-47.47) | 18 | r: -0.298; p: 0.229 | >0.999 |
| Chalder score |  | 27.00 (25.00-30.00) | 56 | r: -0.201; p: 0.138 | >0.999 | 27.00 (25.00-30.25) | 38 | r: -0.287; p: 0.080 | >0.999 | 26.00 (24.00-29.50) | 17 | r: -0.318; p: 0.213 | >0.999 |
| SF-36 score |  | 50.00 (25.00-65.00) | 43 | r: 0.204; p: 0.190 | >0.999 | 50.00 (20.00-65.00) | 31 | r: 0.206; p: 0.265 | >0.999 | 50.00 (27.50-68.75) | 12 | r: 0.358; p: 0.253 | >0.999 |

**Table S4(A)**: Correlations between sCD26 and laboratory assessments (onset stratified)

| parameter | | Whole cohort (n: 205) | | | | Patients with infection-triggered onset (n: 146) | | | | Patients with non-infection-triggered onset (n: 57) | | | |
| --- | --- | --- | --- | --- | --- | --- | --- | --- | --- | --- | --- | --- | --- |
|  |  | median (IQR)/% | n (criteria positive) | sCD26 Correlation/  Mann-U results | BY-corrected p | median (IQR)/% | n (criteria positive) | sCD26 Correlation/  Mann-U results | BY-corrected p | median (IQR)/% | n (criteria positive) | sCD26 Correlation/  Mann-U results | BY-corrected p |
| Immunological assessment:  CrP  IL-1b  soluble IL-2 receptor  Ferritin  Angiotensin converting enzyme  C3 complement  C4 complement  lymphocytes  monocytes  granulocytes  natural killer (NK) cells  CD19+ B cells  CD3+ T cells  CD4+ T cells  CD8+ T cells  CD4+CD8+ T cells  CD4/CD8-ratio  HLA-DR+/CD8+ T cells  CD11a+/CD8- T cells  CD28+/CD8+ T cells  CD57+/CD8- T cells  monocytal HLA-DR expression  ANA >1:160  IgG  IgA  IgM  IgE | mg/dl  pg/ml  IU/ml  µg/ml  U/l  mg/dl  mg/dl  /nl  /nl  /nl  /nl  /nl  /nl  /nl  /nl  %  %  %  %  %  ab/cell  g/l  g/l  g/l  kU/l | 1.60 (0.60-3.00)  212.50 (147-353)  335.00 (277-436)  68.90 (37-110)  26.80 (19-34)  1030 (880-1200)  230 (180-280)  1.88 (1.51-2.26)  0.43 (0.34-0.57)  4.18 (3.17-5.32)  0.18 (0.13-0.27)  0.22 (0.16-0.30)  1.40 (1.11-1.71)  0.88 (0.71-1.13)  0.40 (0.30-0.52)  1.13 (0.75-1.97)  2.20 (1.70-2.80)  12 (6-19)  52 (39-64)  80 (69-88)  2 (1-4)  39917 (33252-47994)  13%  9.72 (8.36-11.06)  1.71 (1.25-2.14)  1.06 (0.72-1.49)  28.10 (11.00-80.30) | 202  170  196  203  167  170  170  183  183  183  183  182  183  183  183  183  183  172  170  172  172  181  173 (23)  203  203  203  195 | r: 0.023; p. 0.740  **r: -0.182; p: 0.018***  r: 0.039; p: 0.586  **r: 0.144; p: 0.041***  r: 0.121; p: 0.119  r: 0.032; p: 0.683  r: 0.007; p: 0.930  **r: -0.168; p: 0.023***  r: -0.086; p: 0.245  r: -0.101; p: 0.172  r: -0.112; p: 0.132  r: 0.001; p: 0.986  **r: -0.180; p: 0.015***  **r: -0.207; p: 0.005***  r: -0.063; p: 0.398  **r: 0.153; p: 0.039***  r: -0.140; p: 0.059  **r: 0.316; p: <0.001***  r: 0.147; p: 0.057  r: -0.131; p: 0.088  r: 0.044; p: 0.569  r: 0.010; p: 0.897  p: 0.707  r: -0.051; p: 0.467  r: <0.001; p: 0.996  r: -0.093; p: 0.186  r: -0.029; p: 0.687 | >0.999  0.396  >0.999  0.642  >0.999  >0.999  >0.999  0.476  >0.999  >0.999  >0.999  >0.999  0.366  0.165  >0.999  0.642  0.837  **0.007***  0.837  >0.999  >0.999  >0.999  >0.999  >0.999  >0.999  >0.999 | 1.80 (0.60-3.00)  202.50 (140-353)  331.50 (278-400)  61.00 (33-106)  26.30 (19-33)  1025 (880-1193)  230 (170-273)  1.87 (1.50-2.26)  0.43 (0.34-0.57)  4.18 (3.15-5.31)  0.18 (0.13-0.27)  0.21 (0.16-0.28)  1.37 (1.10-1.71)  0.86 (0.70-1.11)  0.39 (0.31-0.51)  1.09 (0.72-1.88)  2.20 (1.70-2.80)  10 (6-18)  49 (38-61)  81 (70-88)  2 (1-3)  39236 (32568-48487)  17%  9.78 (8.37-11.15)  1.75 (1.28-2.14)  1.00 (0.70-1.43)  27.20 (10.40-76.30) | 143  118  138  144  115  118  118  129  129  129  129  129  129  129  129  129  129  120  118  120  120  128  121 (20)  144  144  144  139 | r: 0.021; p: 0.804  **r: -0.297; p: 0.001***  r: 0.064; p: 0.453  **r: 0.229; p: 0.006***  r: 0.041; p: 0.660  r: 0.058; p:0.530  r: 0.003; p: 0.978  **r: -0.221; p: 0.012***  r: -0.045; p: 0.612  r: -0.061; p: 0.494  r: -0.100; p: 0.260  r: -0.090; p: 0.312  **r: -0.215; p: 0.004***  **r: -0.227; p: 0.010***  r: -0.115; p: 0.193  r: 0.139; p: 0.116  r: -0.113; p: 0.200  **r: 0.345; p: <0.001***  r: 0.140; p: 0.130  r: -0.151; p: 0.099  r: 0.026; p: 0.774  r: 0.005; p: 0.959  p: 0.494  r: -0.056; p: 0.502  r: 0.138; p: 0.098  **r: -0.179; p: 0.031***  r: 0.050; p: 0.557 | >0.999  **0.035***  >0.999  0.140  >0.999  >0.999  >0.999  0.249  >0.999  >0.999  >0.999  >0.999  0.273  0.218  >0.999  >0.999  >0.999  **0.006***  >0.999  >0.999  >0.999  >0.999  >0.999  >0.999  0.445  >0.999 | 1.50 (0.75-3.00)  265.00 (158-407)  350.00 (256-491)  88.50 (50-131)  27.20 (20-36)  1030 (910-1210)  230 (200-300)  1.98 (1.55-2.28)  0.44 (0.34-0.58)  4.27 (3.24-5.33)  0.19 (0.14-0.28)  0.23 (0.17-0.34)  1.45 (1.14-1.71)  0.96 (0.79-1.19)  0.40 (0.29-0.54)  1.37 (0.84-2.39)  2.40 (1.70-2.90)  14 (7-24)  56 (45-68)  80 (60-90)  2 (1-4)  41362 (34160-48037)  6%  9.59 (8.35-10.46)  1.60 (1.16-2.36)  1.16 (0.75-1.61)  44.90 (12.80-108.80) | 57  51  56  57  51  51  51  52  52  52  52  52  52  52  52  52  52  51  51  51  51  52  51 (3)  57  57  57  55 | r: 0.088; p: 0.516  r: 0.107; p: 0.454  r: -0.047; p: 0.732  r: -0.056; p: 0.680  **r: 0.307; p: 0.028***  r: -0.027; p: 0.851  r: 0.005; p: 0.971  r: -0.065; p: 0.647  r: -0.181; p: 0.200  r: -0.206; p: 0.143  r: -0.082; p: 0.566  **r: 0.301; p: 0.030***  r: -0.104; p: 0.464  r: -0.196; p: 0.164  r: 0.022; p: 0.875  r: 0.208; p: 0.139  r: -0.156; p: 0.269  r: 0.244; p: 0.085  r: 0.217; p: 0.126  r: -0.103; p: 0.472  r: 0.118; p: 0.408  r: 0.059; p: 0.676  p: 0.454  r: 0.024; p: 0.861  **r: -0.285; p: 0.032***  r: 0.145; p: 0.282  r: -0.173; p: 0.207 | >0.999  >0.999  >0.999  >0.999  >0.999  >0.999  >0.999  >0.999  >0.999  >0.999  >0.999  >0.999  >0.999  >0.999  >0.999  >0.999  >0.999  >0.999  >0.999  >0.999  >0.999  >0.999  >0.999  >0.999  >0.999  >0.999  >0.999 |
| Markers of organ function  LDH  CK  HbA1c  GPT  GOT  GGT  Albumin  Bilirubin  Creatinine  Total protein  NTproBNP | U/l  U/l  %  U/l  U/l  U/l  g/l  mg/dl  mg/dl  g/l  ng/l | 231 (207-263)  82.00 (63-112)  5.20 (4.90-5.40)  21.00 (16-29)  24.00 (21-27)  17.00 (12-25)  45.70 (44-47)  0.37 (0.29-0.56)  0.76 (0.68-0.86)  71.00 (69-75)  41.00 (23-73) | 201  195  189  202  202  202  189  195  205  169  190 | **r: 0.173; p: 0.014***  **r: 0.258; p: <0.001***  r: 0.114; p: 0.120  **r: 0.273; p: <0.001***  **r: 0.221; p: 0.002***  **r: 0.262; p: <0.001***  r: 0.121; p: 0.096  r: 0.055; p: 0.447  **r: 0.247; p: <0.001***  r: 0.038; p: 0.628  r: -0.082; p: 0.261 | 0.366  **0.019***  >0.999  **0.012***  0.072  **0.015***  >0.999  >0.999  **0.019***  >0.999  >0.999 | 232 (204-263)  82.00 (63-110)  5.20 (4.90-5.30)  20.00 (15-28)  24.00 (20-27)  15.00 (12-22)  45.70 (44-48)  0.41 (0.29-0.60)  0.77 (0.68-0.85)  71.00 (69-75)  39.50 (23-70) | 142  139  133  143  143  143  133  139  146  116  132 | **r: 0.272; p: 0.001***  **r: 0.353; p: <0.001***  **r: 0.183; p: 0.035***  **r: 0.349; p: <0.001***  **r. 0.336; p: <0.001***  **r: 0.326; p: <0.001***  **r: 0.197; p: 0.023***  r: 0.107; p: 0.210  **r: 0.252; p: 0.002***  r: 0.098; p: 0.116  r: -0.134; p: 0.125 | **0.035***  **0.003***  0.478  **0.003***  **0.004***  **0.005***  0.350  >0.999  0.059  >0.999  >0.999 | 225 (211-264)  86.00 (65-114)  5.20 (5.10-5.50)  22.00 (18-34)  25.00 (22-30)  19.00 (13-29)  45.50 (45-47)  0.32 (0.28-0.48)  0.75 (0.68-0.88)  71.00 (68-73)  45.50 (22-82) | 57  55  55  57  57  57  55  55  57  51  56 | r: -0.088; p: 0.510  r: 0.051; p: 0.712  r: -0.010; p: 0.943  r: 0.109; p: 0.418  r: -0.037; p: 0.787  r: 0.061; p: 0.650  r: -0.155; p: 0.259  r: -0.192; p: 0.160  r: 0.228; p: 0.089  r: -0.119; p: 0.407  r: -0.003; p: 0.984 | >0.999  >0.999  >0.999  >0.999  >0.999  >0.999  >0.999  >0.999  >0.999  >0.999  >0.999 |
| Electrolytes:  Sodium  Potassium  Calcium (Albumin-corrected) ^1^  Phosphate  Zinc | mmol/l  mmol/l  mmol/l  mmol/l  µmol/l | 141.00 (139-142)  3.90 (3.70-4.10)  2.23 (2.17-2.28)  1.00 (0.87-1.11)  13.10 (11.70-14.75) | 201  201  189  193  185 | r: 0.050; p: 0.483  r: -0.064; p: 0.367  r: 0.133; p: 0.069  r: -0.003; p: 0.968  r: 0.113; p: 0.126 | >0.999  >0.999  0.928  >0.999  >0.999 | 141.00 (139-142)  3.90 (3.70-4.10)  2.23 (2.17-2.28)  1.01 (0.87-1.11)  13.00 (11.80-14.70) | 143  143  132  138  131 | r: 0.046; p: 0.587  r: -0.082; p: 0.331  r: 0.135; p: 0.123  r: -0.116; p: 0.175  r; 0.157; p: 0.074 | >0.999  >0.999  >0.999  >0.999  0.948 | 141.00 (139-143)  3.90 (3.70-4.20)  2.23 (2.18-2.27)  0.98 (0.88-1.09)  13.30 (11.63-14.78) | 56  56  55  54  52 | r: 0.058; p: 0.672  r: -0.064; p: 0.639  r: 0.147; p: 0.283  **r: 0.321; p: 0.018***  r: -0.034; p: 0.810 | >0.999  >0.999  >0.999  >0.999  >0.999 |
| Thyroid assessment:  fT3  fT4  TSH basal  Thyroid peroxidase (TPO)-AAB  Thyrotropin receptor (TR)-AAB  Thyroglobulin (Tg)-AAB | ng/l  ng/l  mU/l  kU/l  kU/l  U/l | 3.26 (2.92-3.56)  12.52 (11.65-14.31)  1.46 (1.01-1.99)  9.00 (8.00-12.00)  0.89 (0.89-0.89)  10.40 (10-18) | 103  104  192  107  104  102 | **r: 0.206; p: 0.037***  r: -0.121; p: 0.220  r: 0.064; p: 0.376  r: 0.024; p: 0.806  r: -0.014; p: 0.885  r: 0.045; p: 0.654 | 0.642  >0.999  >0.999  >0.999  >0.999  >0.999 | 3.28 (2.96-3.59)  12.55 (11.66-14.30)  1.50 (1.01-2.04)  9.00 (8.00-12.00)  0.89 (0.89-0.89)  10.00 (10-16) | 68  69  139  71  68  67 | **r: 0.275; p: 0.023***  r: -0.069; p: 0.574  r: 0.069; p: 0.421  r: 0.090; p: 0.454  r: 0.127; p: 0.303  r: 0.024; p: 0.844 | 0.350  >0.999  >0.999  >0.999  >0.999  >0.999 | 3.07 (2.87-3.39)  12.42 (11.52-14.16)  1.26 (0.95-1.81)  9.00 (8.00-15.00)  0.89 (0.89-0.89)  12.80 (10-70) | 33  33  52  34  34  33 | r: 0.007; p: 0.958  r: -0.274; p: 0.123  r: 0.101; p: 0.474  r: -0.130; p: 0.464  r: -0.271; p: 0.121  r: 0.050; p: 0.782 | >0.999  >0.999  >0.999  >0.999  >0.999  >0.999 |
| Autoantibodies:  alpha1-AR-AAB  alpha2-AR-AAB  beta1-AR-AAB  beta2-AR-AAB  beta3 -AR-AAB  M3-mAChR-AAB  M4-mAChR-AAB  AT1-R-AAB  ETA-R-AAB  ETB-R-AAB | U/ml  U/ml  U/ml  U/ml  U/ml  U/ml  U/ml  U/ml  U/ml  U/ml | 7.65 (5.09-10.48)  9.10 (6.89-12.42)  8.71 (6.00-12.63)  6.40 (4.61-9.79)  7.30 (5.12-11.48)  4.42 (3.35-6.09)  7.01 (5.49-9.72)  12.02 (9.44-17.06)  9.73 (7.34-12.43)  13.37 (10.41-17.98) | 200  202  202  204  200  203  203  205  205  199 | **r: -0.172; p: 0.015***  r: 0.011; p: 0.878  **r: -0.155; p: 0.027***  r: -0.111; p: 0.113  r: -0.053; p: 0.456  **r: -0.210; p: 0.003***  r: -0.022; p: 0.752  r: 0.030; p: 0.665  r: 0.025; p: 0.717  r: -0.040; p: 0.576 | 0.366  >0.999  0.524  >0.999  >0.999  0.100  >0.999  >0.999  >0.999  >0.999 | 7.60 (5.05-10.53)  8.85 (6.75-12.28)  8.86 (5.99-13.16)  6.55 (4.62-10.35)  7.37 (5.21-11.51)  4.49 (3.37-6.26)  7.12 (5.49-9.82)  12.07 (9.39-16.91)  9.34 (7.57-12.43)  13.61 (10.59-18.70) | 142  143  143  145  141  145  145  146  146  141 | **r: -0.270; p: 0.001***  r: -0.053; p: 0.527  **r: -0.201; p: 0.016***  **r: -0.198; p: 0.017***  r: -0.082; p: 0.332  **r: -0.304; p: <0.001***  r: -0.118; p: 0.156  r: -0.066; p: 0.427  r: -0.019; p: 0.819  r: -0.078; p: 0.357 | **0.035***  >0.999  0.290  0.290  >0.999  **0.009***  >0.999  >0.999  >0.999  >0.999 | 7.79 (4.61-9.92)  9.21 (7.17-12.67)  8.57 (6.27-11.59)  6.29 (4.61-9.19)  7.07 (4.77-10.53)  4.10 (3.18-5.87)  6.84 (5.27-9.60)  11.74 (9.47-17.13)  9.90 (8.11-12.30)  12.72 (10.25-15.80) | 56  57  57  57  57  56  56  57  57  56 | r: 0.067; p: 0.624  r: 0.126; p: 0.350  r: 0.015; p: 0.913  r: 0.156; p: 0.245  r: 0.072; p: 0.595  r: 0.021; p: 0.879  r: 0.167; p: 0.218  r: 0.243; p: 0.069  r: 0.102; p: 0.451  r: 0.122; p: 0.370 | >0.999  >0.999  >0.999  >0.999  >0.999  >0.999  >0.999  >0.999  >0.999  >0.999 |

^1^ Albumin-correction for Calcium was performed as: Calcium_corrected_ (mmol/l) = Calcium_total_ (mmo/l) – (0.025 x Albumin (g/l)) + 1

**Table S4(B)**: Correlations between sCD26 and Schellong examination outcome (onset stratified)

| parameter | | Whole cohort (n: 205) | | | | Patients with infection-triggered onset (n: 146) | | | | Patients with non-infection-triggered onset (n: 57) | | | |
| --- | --- | --- | --- | --- | --- | --- | --- | --- | --- | --- | --- | --- | --- |
|  |  | median (IQR) | n | sCD26 Correlation results | BY-corrected p | median (IQR) | n | sCD26 Correlation results | BY-corrected p | median (IQR) | n | sCD26 Correlation results | BY-corrected p |
| heart rate (seated)  blood pressure (sys) (seated)  blood pressure (dia) (seated) | /min  mmHg  mmHg | 80 (71-91)  130 (118-144)  88 (81-97) | 182  180  180 | r: -0.030; p: 0.685  **r: 0.159; p: 0.034***  r: 0.110: p: 0.141 | >0.999  0.352  0.875 | 81 (72-92)  128 (117-142)  87 (81-96) | 129  127  127 | r: 0.066; p: 0.459  **r: 0.204; p: 0.022***  r: 0.153; p: 0.085 | >0.999  0.229  0.767 | 78 (69-91)  137 (124-145)  91 (82-98) | 51  51  51 | **r: -0.291; p: 0.038***  r: 0.052; p: 0.717  r: 0.019; p: 0.897 | 0.442  >0.999  >0.999 |
| heart rate standing 0min  heart rate after 2min  heart rate after 5min  heart rate after 10min  blood pressure (sys) standing 0min  blood pressure (dia) standing 0min  blood pressure (sys) after 2min  blood pressure (dia) after 2min  blood pressure (sys) after 5min  blood pressure (dia) after 5min  blood pressure (sys) after 10min  blood pressure (dia) after 10min  changes heartrate seated🡪standing  changes RR (sys) seated🡪standing  changes RR (dia) seated🡪standing | /min  /min  /min  /min  mmHg  mmHg  mmHg  mmHg  mmHg  mmHg  mmHg  mmHg  /min  mmHg  mmHg | 89 (81-98)  91 (81-101)  89 (81-101)  93 (83-103)  128 (117-143)  90 (84-101)  127 (118-143)  92 (85-103)  128 (118-140)  92 (86-100)  125 (114-138)  92 (83-103)  9 (3-14)  -2 (-9-5)  2 (-3-7) | 180  179  177  115  178  178  176  176  177  177  114  115  180  178  178 | **r: -0.148; p: 0.048***  r: -0.103; p: 0.171  r: -0.106; p: 0.162  r: -0.126; p: 0.181  **r: 0.185; p: 0.014***  r. 0.094; p: 0.213  **r: 0.210; p: 0.005***  r: 0.119; p: 0.114  **r: 0.225; p: 0.003***  **r: 0.189; p: 0.012***  r: 0.165; p: 0.079  r: 0.117; p: 0.214  **r: -0.226; p: 0.002***  r: 0.073; p: 0.333  r: -0.008; p: 0.919 | 0.428  0.875  0.875  0.875  0.170  0.898  0.110  0.799  0.081  0.170  0.620  0.898  0.081  >0.999  >0.999 | 89 (81-98)  91 (83-102)  90 (84-101)  93 (86-103)  126 (116-141)  88 (83-100)  125 (117-141)  92 (85-101)  125 (117-140)  92 (86-99)  125 (113-137)  92 (83-103)  9 (3-14)  -2 (-8-5)  2 (-4-7) | 128  126  124  85  126  126  123  123  124  124  85  85  128  126  126 | r: -0.041; p: 0.646  r: 0.001; p: 0.993  r: 0.053; p: 0.559  r: -0.015; p: 0.894  **r: 0.209; p: 0.019***  r: 0.133; p: 0.137  **r: 0.272; p: 0.002***  r: 0.145; p: 0.108  **r: 0.282; p: 0.002***  **r: 0.245; p: 0.006***  r: 0.173; p: 0.114  r: 0.151; p: 0.167  **r: -0.203; p: 0.022***  r: -0.009; p: 0.920  r: -0.018; p: 0.843 | >0.999  >0.999  >0.999  >0.999  0.229  0.860  0.072  0.794  0.072  0.130  0.794  0.957  0.229  >0.999  >0.999 | 89 (74-98)  87 (76-98)  89 (75-98)  93 (80-105)  130 (124-150)  92 (86-104)  131 (121-153)  94 (85-106)  133 (121-149)  95 (85-106)  125 (115-149)  92 (81-103)  9 (4-14)  -2 (-10-6)  3 (-2-8) | 50  51  51  29  50  50  51  51  51  51  28  29  50  50  50 | **r: -0.438; p: 0.001***  **r: -0.366; p: 0.008***  **r: -0.450; p: 0.001***  **r: -0.420; p: 0.023***  r: 0.181; p: 0.208  r: 0.040; p: 0.780  r: 0.091; p: 0.525  r: 0.099; p: 0.490  r: 0.119; p: 0.404  r: 0.059; p: 0.679  r: 0.165; p: 0.401  r: -0.024; p: 0.900  r: -0.256; p: 0.073  **r: 0.289; p: 0.042***  r: 0.003; p: 0.984 | **0.046***  0.172  **0.046***  0.367  >0.999  >0.999  >0.999  >0.999  >0.999  >0.999  >0.999  >0.999  0.652  0.442  >0.999 |

**Table S4(C)**: Correlations between sCD26 and clinical questionnaire outcome (onset stratified)

| parameter | | Whole cohort (n: 205) | | | | Patients with infection-triggered onset (n: 146) | | | | Patients with non-infection-triggered onset (n: 57) | | | |
| --- | --- | --- | --- | --- | --- | --- | --- | --- | --- | --- | --- | --- | --- |
|  |  | median (IQR) | n | sCD26 Correlation results | BY-corrected p | median (IQR) | n | sCD26 Correlation results | BY-corrected p | median (IQR) | n | sCD26 Correlation results | BY-corrected p |
| Symptom scores  fatigue  fatigue score  cognitive score  muscle pain  immune score  disease severity |  | 8.00 (7.00-9.00)  8.25 (7.50-9.13)  7.12 (5.67-8.00)  7.00 (5.00-8.00)  5.33 (3.67-6.67)  8.00 (7.00-8.50) | 186  187  185  185  187  159 | r: -0.008; p: 0.914  r: 0.057; p: 0.435  r: 0.045; p: 0.544  r: -0.030; p: 0.684  r: 0.115; p: 0.116  r: 0.030; p: 0.711 | >0.999  >0.999  >0.999  >0.999  >0.999  >0.999 | 8.00 (7.00-9.00)  8.25 (7.50-9.00)  7.33 (5.67-8.00)  7.00 (5.00-8.00)  5.33 (3.92-7.00)  8.00 (7.00-8.00) | 132  133  131  133  133  112 | r: 0.017; p: 0.845  r: 0.089; p: 0.310  r: 0.002; p: 0.981  r: -0.030; p: 0.728  **r: 0.197; p: 0.023***  r: 0.029; p: 0.760 | >0.999  >0.999  >0.999  >0.999  0.676  >0.999 | 8.25 (8.00-10.00)  8.25 (7.50-9.25)  7.00 (5.75-8.00)  8.00 (6.00-8.00)  5.33 (3.67-6.25)  8.00 (7.00-9.00) | 52  52  52  50  52  45 | r: -0.026; p: 0.852  r: -0.034; p: 0.811  r: 0.160; p: 0.258  r: 0.048; p: 0.740  r: -0.022; p: 0.878  r: 0.075; p: 0.623 | >0.999  >0.999  >0.999  >0.999  >0.999  >0.999 |
| Bell score |  | 30.00 (30.00-40.00) | 202 | r: -0.069; p: 0.332 | >0.999 | 30.00 (30.00-40.00) | 143 | r: -0.131; p: 0.119 | >0.999 | 30.00 (30.00-40.00) | 57 | r: 0.152; p: 0.260 | >0.999 |
| COMPASS 31 score total |  | 44.81 (33.75-53.16) | 192 | r: -0.001; p: 0.994 | >0.999 | 44.20 (33.62-52.65) | 136 | r: 0.037; p: 0.672 | >0.999 | 46.56 (38.09-55.45) | 54 | r: -0.061; p: 0.654 | >0.999 |
| Chalder score |  | 27.00 (25.00-30.00) | 192 | r: -0.053; p: 0.464 | >0.999 | 27.00 (25.00-30.00) | 137 | r: -0.062; p: 0.474 | >0.999 | 27.00 (24.00-30.00) | 53 | r: -0.070; p: 0.617 | >0.999 |
| SF-36 score |  | 37.50 (20.00-55.00) | 136 | r: 0.031; p: 0.721 | >0.999 | 35.00 (17.50-57.50) | 93 | r: -0.032; p: 0.764 | >0.999 | 40.00 (20.00-55.00) | 42 | r: 0.165; p: 0.297 | >0.999 |

**References (Figure S3):**

1. Hartwig J, Sotzny F, Bauer S, Heidecke H, Riemekasten G, Dragun D, et al. Research article IgG stimulated β2 adrenergic receptor activation is attenuated in patients with ME/CFS. *Brain, Behavior, & Immunity - Health* (2020):100047. doi: 10.1016/j.bbih.2020.100047.

2. Grisanti LA, Perez DM, Porter JE. Modulation of immune cell function by alpha(1)-adrenergic receptor activation. *Curr Top Membr* (2011) 67:113-38. Epub 2011/07/21. doi: 10.1016/B978-0-12-384921-2.00006-9. PubMed PMID: 21771488; PubMed Central PMCID: PMCPMC3624728.

3. Lettau M, Dietz M, Vollmers S, Armbrust F, Peters C, Dang TM, et al. Degranulation of human cytotoxic lymphocytes is a major source of proteolytically active soluble CD26/DPP4. *Cell Mol Life Sci* (2019). Epub 2019/07/14. doi: 10.1007/s00018-019-03207-0. PubMed PMID: 31300870.

4. Oliveira BC, Marques VB, Brun BF, de Oliveira ESHM, Freitas Soares Melo S, Oliveira EM, et al. Dipeptidyl peptidase-4 inhibition prevents vascular dysfunction induced by beta-adrenergic hyperactivity. *Biomed Pharmacother* (2019) 113:108733. Epub 2019/03/13. doi: 10.1016/j.biopha.2019.108733. PubMed PMID: 30861410.

5. Ikushima H, Munakata Y, Iwata S, Ohnuma K, Kobayashi S, Dang NH, et al. Soluble CD26/dipeptidyl peptidase IV enhances transendothelial migration via its interaction with mannose 6-phosphate/insulin-like growth factor II receptor. *Cell Immunol* (2002) 215(1):106-10. Epub 2002/07/27. PubMed PMID: 12142042.

6. Fryer RM, Segreti J, Banfor PN, Widomski DL, Backes BJ, Lin CW, et al. Effect of bradykinin metabolism inhibitors on evoked hypotension in rats: rank efficacy of enzymes associated with bradykinin-mediated angioedema. *Br J Pharmacol* (2008) 153(5):947-55. Epub 2007/12/18. doi: 10.1038/sj.bjp.0707641. PubMed PMID: 18084312; PubMed Central PMCID: PMCPMC2267285.

7. Vanderheyden M, Bartunek J, Goethals M, Verstreken S, Lambeir AM, De Meester I, et al. Dipeptidyl-peptidase IV and B-type natriuretic peptide. From bench to bedside. *Clin Chem Lab Med* (2009) 47(3):248-52. Epub 2009/03/31. doi: 10.1515/CCLM.2009.065. PubMed PMID: 19327104.

8. Mason RP, Jacob RF, Kubant R, Ciszewski A, Corbalan JJ, Malinski T. Dipeptidyl peptidase-4 inhibition with saxagliptin enhanced nitric oxide release and reduced blood pressure and sICAM-1 levels in hypertensive rats. *J Cardiovasc Pharmacol* (2012) 60(5):467-73. Epub 2012/08/31. doi: 10.1097/FJC.0b013e31826be204. PubMed PMID: 22932707.

9. Kim M, Platt MJ, Shibasaki T, Quaggin SE, Backx PH, Seino S, et al. GLP-1 receptor activation and Epac2 link atrial natriuretic peptide secretion to control of blood pressure. *Nat Med* (2013) 19(5):567-75. Epub 2013/04/02. doi: 10.1038/nm.3128. PubMed PMID: 23542788.

10. Raj SR, Biaggioni I, Yamhure PC, Black BK, Paranjape SY, Byrne DW, et al. Renin-aldosterone paradox and perturbed blood volume regulation underlying postural tachycardia syndrome. *Circulation* (2005) 111(13):1574-82. Epub 2005/03/23. doi: 10.1161/01.CIR.0000160356.97313.5D. PubMed PMID: 15781744.

11. Raschke S, Eckardt K, Bjorklund Holven K, Jensen J, Eckel J. Identification and validation of novel contraction-regulated myokines released from primary human skeletal muscle cells. *PLoS One* (2013) 8(4):e62008. Epub 2013/05/03. doi: 10.1371/journal.pone.0062008. PubMed PMID: 23637948; PubMed Central PMCID: PMCPMC3634789.

12. Nacul L, de Barros B, Kingdon CC, Cliff JM, Clark TG, Mudie K, et al. Evidence of Clinical Pathology Abnormalities in People with Myalgic Encephalomyelitis/Chronic Fatigue Syndrome (ME/CFS) from an Analytic Cross-Sectional Study. *Diagnostics (Basel)* (2019) 9(2). Epub 2019/04/13. doi: 10.3390/diagnostics9020041. PubMed PMID: 30974900; PubMed Central PMCID: PMCPMC6627354.

13. Doupis J, Veves A. DPP4 inhibitors: a new approach in diabetes treatment. *Adv Ther* (2008) 25(7):627-43. Epub 2008/07/22. doi: 10.1007/s12325-008-0076-1. PubMed PMID: 18641927.

14. Kanazawa I, Tanaka K, Sugimoto T. DPP-4 inhibitors improve liver dysfunction in type 2 diabetes mellitus. *Med Sci Monit* (2014) 20:1662-7. Epub 2014/09/18. doi: 10.12659/MSM.890989. PubMed PMID: 25228119; PubMed Central PMCID: PMCPMC4173801.

15. Haluzik M, Frolik J, Rychlik I. Renal Effects of DPP-4 Inhibitors: A Focus on Microalbuminuria. *Int J Endocrinol* (2013) 2013:895102. Epub 2013/10/04. doi: 10.1155/2013/895102. PubMed PMID: 24089613; PubMed Central PMCID: PMCPMC3780627.
